# mRNA maturation in giant viruses: variation on a theme

**DOI:** 10.1093/nar/gkv224

**Published:** 2015-03-16

**Authors:** Stéphane Priet, Audrey Lartigue, Françoise Debart, Jean-Michel Claverie, Chantal Abergel

**Affiliations:** 1 Architecture et Fonction des Macromolécules Biologiques, CNRS UMR 7257, Aix-Marseille Université, 163 Avenue de Luminy, Case 932, 13288 Marseille cedex 9, France; 2 Structural and Genomic Information Laboratory, UMR 7256 (IMM FR 3479) CNRS Aix-Marseille Université, 163 Avenue de Luminy, Case 934, 13288 Marseille cedex 9, France; 3 IBMM, UMR 5247, CNRS-UM1-UM2, Université Montpellier 2, Place Eugène Bataillon, 34095 Montpellier, France; 4 APHM, FR-13385 Marseille, France

## Abstract

Giant viruses from the Mimiviridae family replicate entirely in their host cytoplasm where their genes are transcribed by a viral transcription apparatus. mRNA polyadenylation uniquely occurs at hairpin-forming palindromic sequences terminating viral transcripts. Here we show that a conserved gene cluster both encode the enzyme responsible for the hairpin cleavage and the viral polyA polymerases (vPAP). Unexpectedly, the vPAPs are homodimeric and uniquely self-processive. The vPAP backbone structures exhibit a symmetrical architecture with two subdomains sharing a nucleotidyltransferase topology, suggesting that vPAPs originate from an ancestral duplication. A Poxvirus processivity factor homologue encoded by Megavirus chilensis displays a conserved 5′-GpppA 2′O methyltransferase activity but is also able to internally methylate the mRNAs’ polyA tails. These findings elucidate how the arm wrestling between hosts and their viruses to access the translation machinery is taking place in Mimiviridae.

## INTRODUCTION

The discovery of Mimivirus 10 years ago brought in the realization that DNA viruses could overlap with the cellular world in terms of genome size and complexity (1). Many additional members of the Mimiviridae family have then been isolated from various environments (2,3). To date there are seven fully sequenced representatives of the mimiviruses sub-family, all infecting Acanthamoeba, including Mimivirus (1), Mamavirus (2), Moumouvirus (4), Terra1-2 (5), Megavirus chilensis (6) and Megavirus Iba (7). More distant relatives, belonging to the Mimiviridae family, but infecting other unicellular eukaryotes, have also been sequenced, including Cafeteria roenbergensis virus (CroV, (8)), Phaeocystis globosa virus (PgV, (9)) and the Organic lake phycodnaviruses (OLPV1-2, (10)). They all share an AT-rich (>70%) linear DNA genome with size up to 1.26 Mb encoding up to 1120 proteins, which include a full replication and transcription apparatus. Nucleocytoplasmic Large DNA viruses (NCLDV) (11) comprise the Iridoviridae and the Phycodnaviridae exhibiting mixed nuclear and cytoplasmic replication stages, and exclusively cytoplasmic viruses such as the Poxviridae and the Asfarviridae in addition to the Mimiviridae. Cytoplasmic NCLDV develop a transitory organelle, the virion factory, where all replication processes take place (12–15). The simultaneous delivery from the virion of the fully virally-encoded transcription apparatus together with the genomic DNA, allows the immediate transcription of the early expressed viral genes independently from the host machinery (13,16–20). As expected, all the Mimiviridae infecting Acanthamoeba (belonging to the mimiviruses sub-family) share the same transcriptional regulatory elements. This includes a highly conserved promoter element (‘AAAATTGA’) associated to the early-expressed genes and a more degenerate promoter element associated with the late-expressed genes (18,21). Their genes also present a unique polyadenylation signal that is defined by a hairpin-forming palindromic motif, not conserved at the nucleotide sequence level, instead of the consensus sequence used by eukaryotes and their viruses (6,18,22). This implies that the most distant members of the mimiviruses sub-family, Mimivirus and M. chilensis, despite their significant differences (encoding 50% orthologous proteins that are 50% identical in average), have in common the machinery responsible for the hairpin recognition and polyadenylation. Accordingly, their genomes exhibit a conserved cluster of three genes (Mimivirus R341-R343 and M. chilensis Mg559-561, Supplementary Figure S1A) encoding a remote homologue of the poxviruses polyA polymerase (PAP), a protein of unknown function, and a predicted dsRNA-specific RNAse III, which could be responsible for the 3′-end maturation of the viral transcripts (Supplementary Figure S1C, steps 5 and 6).

Cellular PAPs are classified in two groups. Canonical PAPs have a tripartite structure involving a N-terminal nucleotidyltransferase (NT) catalytic domain, corresponding to the palm domain of polymerases, a central domain, corresponding to the finger domain, and a C-terminal domain corresponding to the RNA binding domain (RBD) (23,24). The only known 3D structure of a non-canonical PAP is that of the mitochondrial PAPD1, which is dimeric and possesses a N-terminal RBD domain (25). The only known structure of a viral PAP is that of Vaccinia virus VP55, and is very different from that of cellular PAPs (26). This structure is nevertheless reminiscent of the non-canonical cellular PAP with a N-terminal domain predicted as the RBD and a central domain corresponding to the catalytic subunit containing the NT domain.

Poxviruses and asfarviruses recognize a sequential signal for transcription termination corresponding to stretches of thymidines in the 3′ untranslated regions (UTR) of the genes (27,28). In addition, polyadenylation in poxviruses requires stretches of uridines at specific positions with respect to the 3′-end of the transcripts (26,29). *In vitro*, the monomeric Vaccinia virus PAP produces polyA tails as short (∼polyA_30_) as that of asfarvirus mRNAs (16) while their mRNA polyA tails are much longer (∼polyA_300_) *in vivo*. This difference in length is due to the processivity factor VP39, which forms a heterodimer with the VP55 PAP (30). Since a homolog of this processivity factor is encoded by M. chilensis genome (Mg18) but is absent from Mimivirus, we determined the crystallographic structure of their PAPs and performed their enzymatic characterization in the presence and absence of the Mg18 protein. We also identified the minimal machinery required to perform the recognition of the hairpin, its cleavage and the mRNA polyadenylation.

Mimivirus and M. chilensis encode a trifunctional mRNA capping enzyme (Mimivirus R382 and M. chilensis Mg512) responsible for capping (Supplementary Figure S1C, step 1) and cap N7 methylation (Supplementary Figure S1C, step 2) (31). Strikingly, the corresponding genes are both located right next to a conserved gene (Mimivirus R383 and M. chilensis Mg511) predicted to encode a 2′O methyltransferase (MTase) conserved in all members of the mimiviruses sub-family (Supplementary Figure S1B). Both the mRNA capping enzyme and the predicted 2′O MTase are present in the virion proteomes (14,17) and could thus be responsible for the 5′-end mRNA maturation (Supplementary Figure S1C, steps 1–3). An unusual cap modification by an additional MTase (L320, conserved in M. chilensis, Mg584) had also been reported for Mimimivirus, which produces a 2,7-dimethylguanosine DMG cap (Supplementary Figure S1C, step 4, (32)). Interestingly, the Vaccinia virus VP39 PAP processivity factor is also a cap 2′O MTase (33). This led us to study the RNA methyltransferase activity and specificity of its homologue in M. chilensis, Mg18.

## MATERIALS AND METHODS

### Plasmids and proteins production

The M. chilensis Mg561 protein (H_6_c-Mg561) was produced as previously described (34). Two constructs have been produced for the Mimivirus R341 gene, which was first amplified from Mimivirus genomic DNA. It was cloned using the Gateway system (Invitrogen) in the pDEST17 expression plasmid to be expressed with a N-terminal His_6_-tag (H_6_+R341). It was also inserted by restriction/ligation in an ‘in house’ modified pETDuet expression vectors (Novagen) allowing the expression with a N-terminal His_6_-Tag (H_6_c+R341). A human rhinovirus 3C protease cleavage site allows tag removal. We refer to the Mimivirus and Megavirus PAPs after tag removal as H_6_c-R341 and H_6_c-Mg561, respectively. The ΔD1 mutants lacking residues 1–43 were generated through site-directed mutagenesis by polymerase chain reaction (PCR) and were named ΔD1 H_6_c+Mg561 (M. chilensis) and ΔD1 H_6_c+R341 (Mimivirus), respectively. Expressions were performed in *E. coli* Rosetta (DE3) by overnight induction at 17°C with 0.2 mM IPTG after OD_600_ reached 0.8. The cultures were left overnight at 17°C. Protein extraction from *E. coli* cells were performed by sonication. The proteins were purified using HisPur Ni-NTA Column (Pierce) washed with Buffer A (50 mM Tris-HCl, 300 mM NaCl buffer, pH 8.5), and then washed with Buffer A containing 25 mM and 50 mM imidazole. Bound proteins were eluted with 500 mM imidazole in Buffer A. After cleavage and purification, the H_6_c-R341 and ΔD1 H_6_c-R341 proteins were dialyzed in CHES 10 mM pH 9 and the H_6_c-Mg561 and ΔD1 H_6_c-Mg561 proteins were dialyzed against Tris-HCl 10 mM pH 9, NaCl 100 mM, prior to concentration using a Amicon-Ultracell 30K centrifugal filter device (Millipore).

The Mg18 and R343 genes were amplified from M. chilensis and Mimivirus genomic DNA and inserted in the modified pETDuet and pDEST42 vectors, for expression of N- and C-terminally tagged proteins, respectively. Expression, protein extraction and purification conditions were the same as for the R341 proteins but using for purification in place of buffer A (above), buffer B (50 mM Tris–HCl, 300 mM NaCl buffer pH 7.5) for Mg18 and buffer C (50 mM Tris-HCl, 300 mM NaCl buffer pH 7.0) for R343. The His_6_- tags were not removed from Mg18 and R343 and the H_6_c+Mg18 and R343+H_6_ proteins were then concentrated using a Amicon-Ultracell 30K centrifugal filter device (Millipore).

The Human N7 methyltransferase (hN7 MTase) protein was expressed, extracted and purified as previously described (35).

### Polyadenylation assay

A synthetic 20mer RNA substrate (5′-GUCCACGUAGACUAACAACU-3′, Biomers) was 5′ end-labeled with T4 polynucleotide kinase (NEB) in the presence of γ-^32^P ATP and purified through Microspin G25 columns (GE healthcare). Polyadenylation reactions were carried out at 30°C in PAP reaction buffer (50 mM Tris pH 7.5, 10 mM KCl, 5 mM DTT) with 500 nM of the ^32^P labeled RNA and 50 nM H_6_c-R341 or H_6_c-Mg561 and were initiated by addition of 5 mM MgCl_2_ or 0.5 mM MnCl_2_ and 100 μM ATP. Where indicated, 100 nM of Mg18 were added to the reaction. To test the nucleotide specificity, ATP was replaced by CTP, GTP or UTP. Reaction products were separated on 8 M urea-14% polyacrylamide gel, and were visualized with a Storm PhosphorImager (Fujifilm) and Image-Gauge software.

### Binding assay

Binding experiments were conducted by biolayer interferometry using a Blitz instrument (FortéBio). For binding analysis of Mg561 with Mg18, Ni-NTA-coated biosensors (ForteBio) were loaded with the H_6_c+Mg18 at 20 μg.ml^−1^ in association buffer (PBS 1X, 0.05% Tween-20, 100 μg.ml^−1^ BSA). After equilibration in association buffer to establish a baseline, a series of Mg561 concentrations ranging from 1.685 to 13.48 μM were ran over the immobilized Mg18 protein in association buffer. The association and dissociation binding traces were recorded at room temperature. The ability of R341 to bind Mg18 was also measured using the H_6_c-R341 protein at 6.0 μM.

### Protein crystallization, structure determination and analysis

For the Mg561 structure determination, protein crystallization and data collection were performed according to (34). The phases were calculated using autoSHARP (36) on a three wavelengths MAD data set in the 51.3–2.24 Å resolution range, and a single solution was found with 34 selenium atoms (for 36 methionines) with a mean figure of merit of 0.39. The solvent flattened electron-density map allowed to automatically build most of the two monomers. The model was refined using AutoBUSTER (37). The final round of refinement resulted in a final *R*_work_ 18.4% and *R*_free_ 21.2%. The refined structure consists of residues 4 to 514 and 516 for monomer A and B, respectively. Residues 263–275 of monomer A and 263–277 of monomer B being disordered in the crystal structure are absent of the final model. 95.4% of the residues are in the most favored regions of the Ramachandran plot and 4.2% are in the additionally allowed regions. Refinement statistics are listed in Table S1.

Crystals of H_6_c-R341 were obtained in 7% PEG 400 (v/v) and 50 mM Imidazole pH 6.4. The H_6_c-R341 was crystallized at 20°C by hanging-drop vapor diffusion using 24-well culture plates (Greiner). Each hanging drop was prepared by mixing 0.5 μl of H_6_c-R341 (1.5 g/l) with 0.3 μl of reservoir solution. The hanging drop on the cover glass was vapor-equilibrated against 1 ml reservoir solution in each well. To improve the crystal diffraction, a previously described evaporation protocol was used where crystals were soaked for 15min in 10 μl reservoir solution containing 10% ethylene glycol as a cryoprotectant (38). Crystals were then flash-frozen at −173°C. Data collection was carried out at −173°C on ID29 beamline at the European Synchrotron Radiation Facility (Grenoble, France). Diffraction intensities were integrated with XDS (39). The structure of R341 was solved by molecular replacement with Phaser (40) using a Mg561 monomer as search model and refined using AutoBUSTER (37). The final round of refinement resulted in a final *R*_work_ 22.7% and *R*_free_ 24.6%. The refined structure consists of residues 9 to 528 and 527 for monomer A and B, respectively. Residues 262–274 and 361–372 of monomer A and 262–276 and 360–368 of monomer B being disordered in the crystal structure are absent of the final model. The quality of the model was validated using Molprobity. 90.8% of the residues are in the most favored regions of the Ramachandran plot and 8.6% are in the additionally allowed regions. Refinement statistics are listed in Table S1.

### Structure analysis

The contact areas between the two Mg561 molecules in the asymmetric unit were analyzed with the PISA server (41). Electrostatic surfaces were calculated with APBS (42).

### *In vitro* synthesis of Megavirus and Mimivirus RNA transcripts

DNA templates for the synthesis of 3′ hairpin-containing RNA transcripts of the M. chilensis Mg592 gene and Mimivirus R418 gene were generated by PCR using as template the M. chilensis genomic DNA (6) or a plasmid containing the R418 gene previously described (43), using a 5′ primer containing the T7 promoter and a 3′ primer complementary to the sequence downstream of the 3′ hairpin. The PCR products corresponded to the full length Mg592 gene (394nt) and the 80nt 3′-end of the R418 gene containing the canonical hairpin in which the natural transcripts are polyadenylated followed by a shorter non-canonical hairpin never used *in vivo*. The RNA transcripts were synthesized *in vitro* at 37°C overnight in 250 μl reactions containing 40 mM Tris-HCl pH 8.0, 5 mM DTT, 2 mM spermidine, 0,01% Triton X-100, 4% PEG8000 (w/v), 8 mM NTPs, 40 mM MgCl_2_, 2.5 U of yeast inorganic pyrophosphatase, 200 nM of DNA template and 100 nM of T7 RNAP (prepared in-house). DNA templates were then digested by 25U of DNase I (Roche). The Mg592 RNA transcripts were purified on 5% acrylamide-urea gel and the R418 ones were purified using the RNeasy mini kit (Qiagen) following the manufacturer's protocol. All RNAs were quantified spectrophotometrically.

### RNase assay

The R418 RNA transcripts were 5′ dephosphorylated using the FastAP phosphatase (Thermo) and then 5′-end-^32^P labeled with T4 polynucleotide kinase (NEB) in the presence of γ-^32^P ATP and purified through Microspin G25 columns (GE healthcare). RNase assay was carried out at 30°C in 50 mM Tris pH 7.5, 150 mM KCl, 5 mM DTT with 250 nM of the R418 ^32^P-labeled RNA transcripts and 1 μM R343+H_6_ in the presence of 5 mM MgCl_2_ or 0.5 mM MnCl_2_. Where indicated, 1 μM of H6c-R341 was added to the reaction. Reaction products were separated on 8 M urea-10% polyacrylamide gel.

### Polyadenylation of mg592 natural transcripts and synthesis of the dscDNA for sequencing

RNA transcripts (1 μg) were *in vitro* polyadenylated at 30°C for 1 hour in PAP reaction buffer, 1 mM ATP, 5 mM MgCl_2_, 0.5 mM MnCl_2_, and 0.5 μM of H_6_c-Mg561 or H_6_c-R341. Control reactions with 2U of bacterial PAP (NEB) were also performed. Where indicated the RNAs were pre-incubated 3 hours with 1 μM of R343+H_6_ and 5 mM MgCl_2_ or 0.5 mM MnCl_2_. Polyadenylated RNA were purified using the Dynabeads mRNA Purification Kit (Invitrogen). PolyA^+^ RNA (150ng) were reverse transcribed using the SMARTScribe reverse transcriptase (Clontech) and a modified oligo(dT) primers (5′-AAGCATTATGCGGCCGCATTCTAGAGGCCGAGGCGGCCGACATGTTTTTTTTTTTTTTTTTTTTTTTTTTTTTTVN-3′, where V corresponds to the mix of A, G, C or T and N to any nucleotides) according to the manufacturer's protocol. Second strand synthesis was performed using the NEBNext mRNA Second Strand Synthesis mix (NEB). Double-strand cDNAs were amplified using GoTaq G2 Flexi DNA Polymerase (Promega) and a specific 5′-primer (5′-CCAGGGGCCCGGATCCATGTCATTTGATTGGGGAGTTAGCCATG-3′) and the modified oligo(dT) 3′-primer. PCR products were sequenced using the specific 5′-primer ordered from the sequencing company (Eurofins).

### Methyltransferase (MTase) Assay

The 5′-triphosphate and capped 13-mers RNA used in this assay were described previously (44): pppAN_13_ (pppAGUUGUUAGUCUA), GpppAN_13_ (GpppAGUUGUUAGUCUA), ^7Me^GpppAN_13_ (^7Me^GpppAGUUGUUAGUCUA) and GpppA_2’OMe_N_13_ (GpppA_2’OMe_GUUGUUAGUCUA). Homopolymeric poly (A), (U), (C) and (G) were purchased from Sigma. *A. castellanii* total RNAs were extracted using the RNeasy Midi kit (Qiagen). PolyA+ mRNAs were then purified using Dynabeads mRNA purification kit (Invitrogen). MTase activity assays were performed at 30°C in 40 mM Tris-HCl pH 7.5, 1 mM DTT, 1 mM MgCl_2_, 0.7 μM RNA substrates, 10 μM AdoMet (NEB), and 0.03 mCi/ml ^3^H-AdoMet (GE Healthcare). H_6_c+Mg18, H_6_c-Mg561, H_6_c-R341, H_6_c+Mg18 + H_6_c-Mg561 or H_6_c-R341 were added to final concentrations of 1 μM. As a control, 0.5 μM of hN7 MTase or 10U of the Vaccinia virus VP39 (NEB) were used. The amounts of ^3^H-CH_3_ transferred onto RNA substrates were determined by using a DEAE filter-binding assay after overnight incubation and scintillation counting.

## RESULTS

### Common features of the viral PolyA polymerases

**Figure 1.**
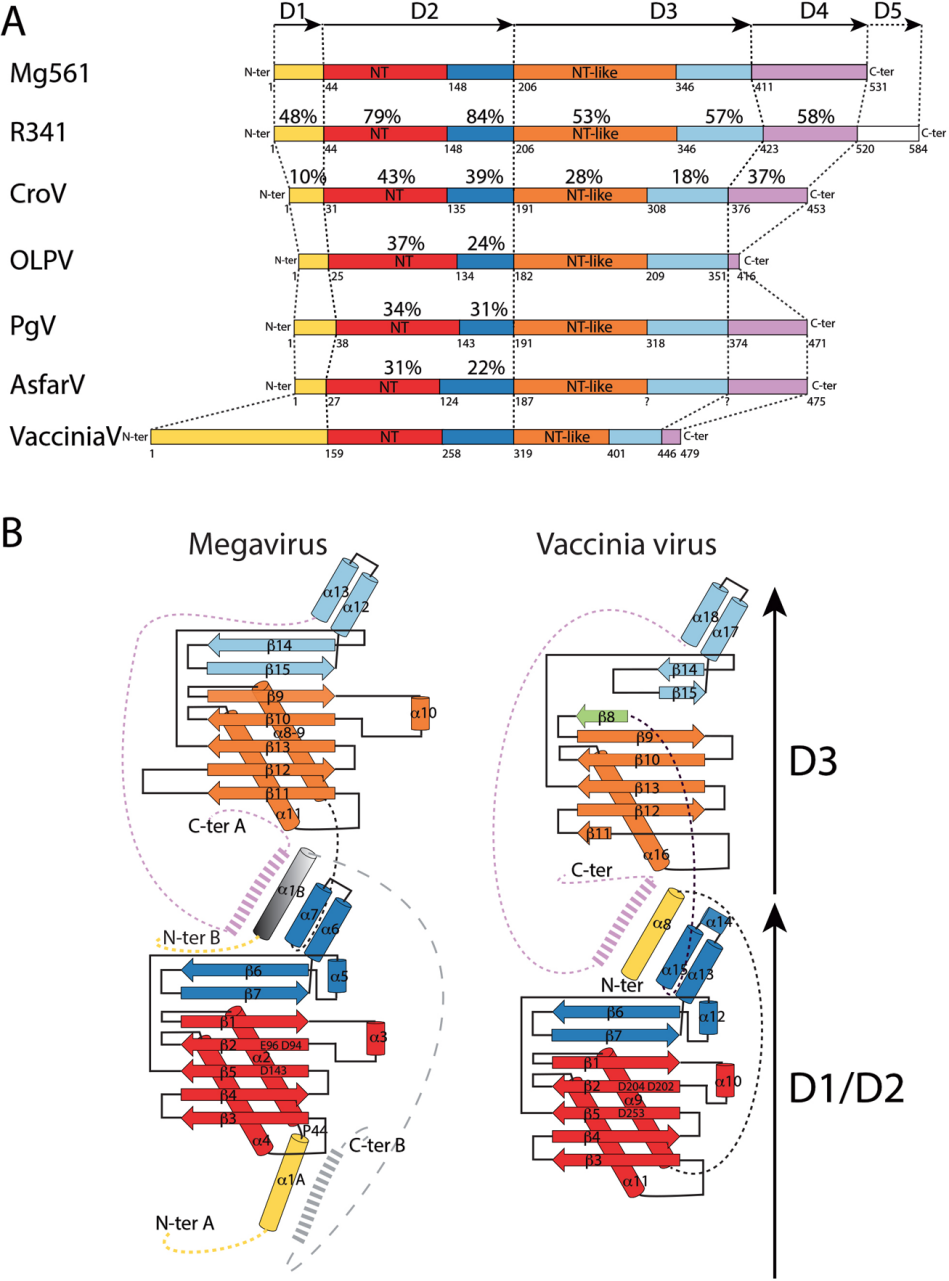
Domain comparison of the viral PolyA polymerases. (**A**) Schematic representation of the different domains found in viral PAPs. The arrows delineate the D1–D5 domains. (**B**) Topology diagram comparing the M. chilensis and Mimivirus PAPs (left panel) with VP55 (right panel, PDB 2GA9). Alpha helices are represented as cylinders and beta strands as arrows. The same color coding is used in A and B, and Supplementary Figure S2. The N-terminal domain D1 is in yellow, the NT domain in red and its terminal extension in blue define the D2 domain, the NT-like in orange and its terminal extension in cyan define the D3 domain, the D4 domain is in purple and the Mimivirus R341 C-terminal extension D5 is in white. When meaningful, the percentage of sequence identity between the various domains relative to the Mg561 is marked on top of each domain.

The Mimivirus R341 and M. chilensis Mg561 genes were reliably predicted to encode PAPs after two iterations of PSI-BLAST (45) (Figure 1 and Supplementary Figure S2). They present the conserved NT signature hG[G/S]x_n_Dh[D/E]h with n = 23 instead of 13 for the Vaccinia virus PAP (46). Their sequences share 62% identity over their entire length, more than the average level of conservation (50%) between orthologous proteins of the two viruses (6,47). The level of sequence conservation between the four subdomains is variable, from 48% identical residues in the N-terminal domain (D1), up to 80% for the catalytic domain (D2) (Figure 1). The Mimivirus R341 protein exhibits a unique acidic extension of 65 residues at its C-terminal end (D5) and an insertion of 12 amino acids in its third subdomain (D3). The sequence comparison of the NCLDV PAPs showed that the D1 domain of Mimivirus and M. chilensis PAPs is unique, while the D3 and D4 domains are conserved in CroV (Crov286). The homology seems to be restricted to the D2 domain with all other viral PAPs.

### R341 and Mg561 gene products are bona fide polyA polymerases

**Figure 2.**
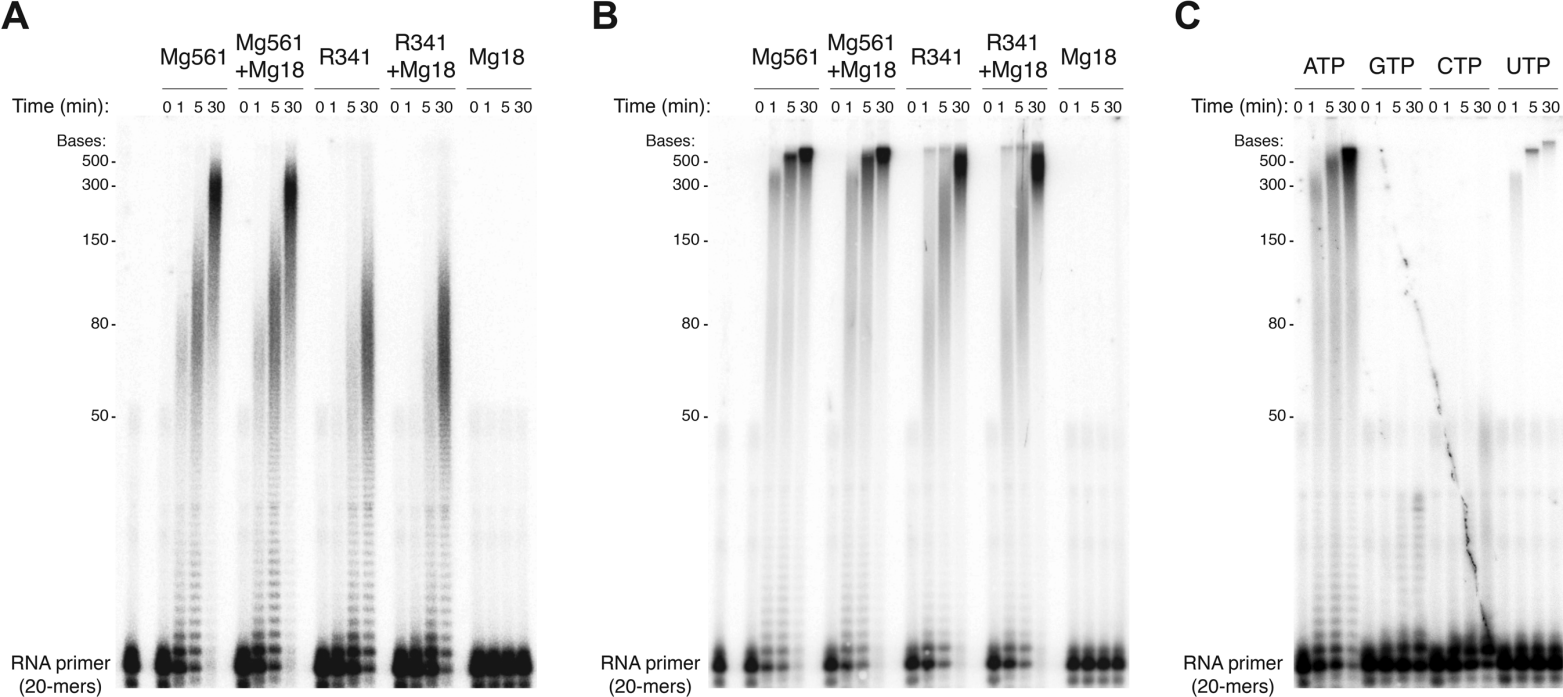
Mg561 and R341 are processive PolyA polymerases. (**A**) Time course (in minutes) PAP assay of Mg561, R341, Mg18 and a mixture of Mg18 with Mg561 or R341, on a 20-mers 5′-^32^P-labeled RNA primer with Mg^2+^ as catalytic ions. (**B**) As in (A), with Mn^2+^ instead of Mg^2+^ (**C**) Catalytic activity of Mg561 on each four nucleotides on a 20-mers and Mn^2+^.

To confirm the functional prediction of Mg561 and R341, the proteins were purified and assayed in an *in vitro* polyadenylation assay on a synthetic 20-mers RNA without secondary structure in the presence of ATP and Mg^2+^ or Mn^2+^ as catalytic ions (Figure 2A and B). The two proteins were able to synthesize long products, apparently proccessively, derived from the RNA and ATP, in the presence of each cation, demonstrating the intrinsic property of Mg561 and R341 to polyadenylate RNA in absence of any specific signal (Supplementary Figure S1C, step 6). This in turn suggested that *in vivo* the hairpin recognition and processing could require additional proteins (Supplementary Figure S1C, step 5). In contrast to the Vaccinia and ASFV PAPs which stop after the addition of ∼30 adenylates (16,48), Mg561 and R341 were able to add much longer polyA tails (up to 700 adenylates). The nucleotide specificity of the PAPs was then assayed in the presence of Mn^2+^ and the 4 NTPs (Figure 2C). As expected, ATP was by far the most efficient nucleotide for homopolymeric tail synthesis. Similar results were obtained when using Mg^2+^ instead of Mn^2+^ (data not shown). However, as observed for non-canonical cellular PAPs known as terminal uridyltransferases (TUTase) or polyU polymerases (PUP) (49), Mg561 was also able to add long polyU tails, although with a reduced activity. This property was also reported for Vaccinia VP55 (29). This enzyme requires uridylates 30–40 nt upstream the polymerization site to be able to proceed further. As a consequence, the enzyme terminates polyadenylation after 30–40 nt and to add long poly(A) tails VP55 needs its processivity factor VP39 (30).

### Is Mg18 a bona fide processivity factor?

We produced the processivity factor homolog Mg18 encoded by the M. chilensis genome, and assayed its binding to Mg561 (Supplementary Figure S3). As expected, Mg18 interacted with Mg561 (*K*_D_ = 6.2 μM), suggesting that it could play a role similar to that of VP39 in the Vaccinia virus heterodimeric polyadenylation complex. No association was observed between Mg18 and R341 confirming the specificity of the interaction. To further investigate the role of Mg18, we tested the Mg561-Mg18 complex in our polyadenylation assay (Figure 2A and B). The presence of Mg18 did not increase the length of the polyA tail nor the polyadenylation velocity, showing that the Mimivirus and M. chilensis enzymes were unaffected by the addition of Mg18, in contrast to eukaryotic and other viral PAPs. Therefore, Mg18 and VP39 are not functionally equivalent and the absence of a Mg18 homolog in Mimivirus has no consequence on the polyadenylation process.

### Structure of the vPAP enzymes

**Figure 3.**
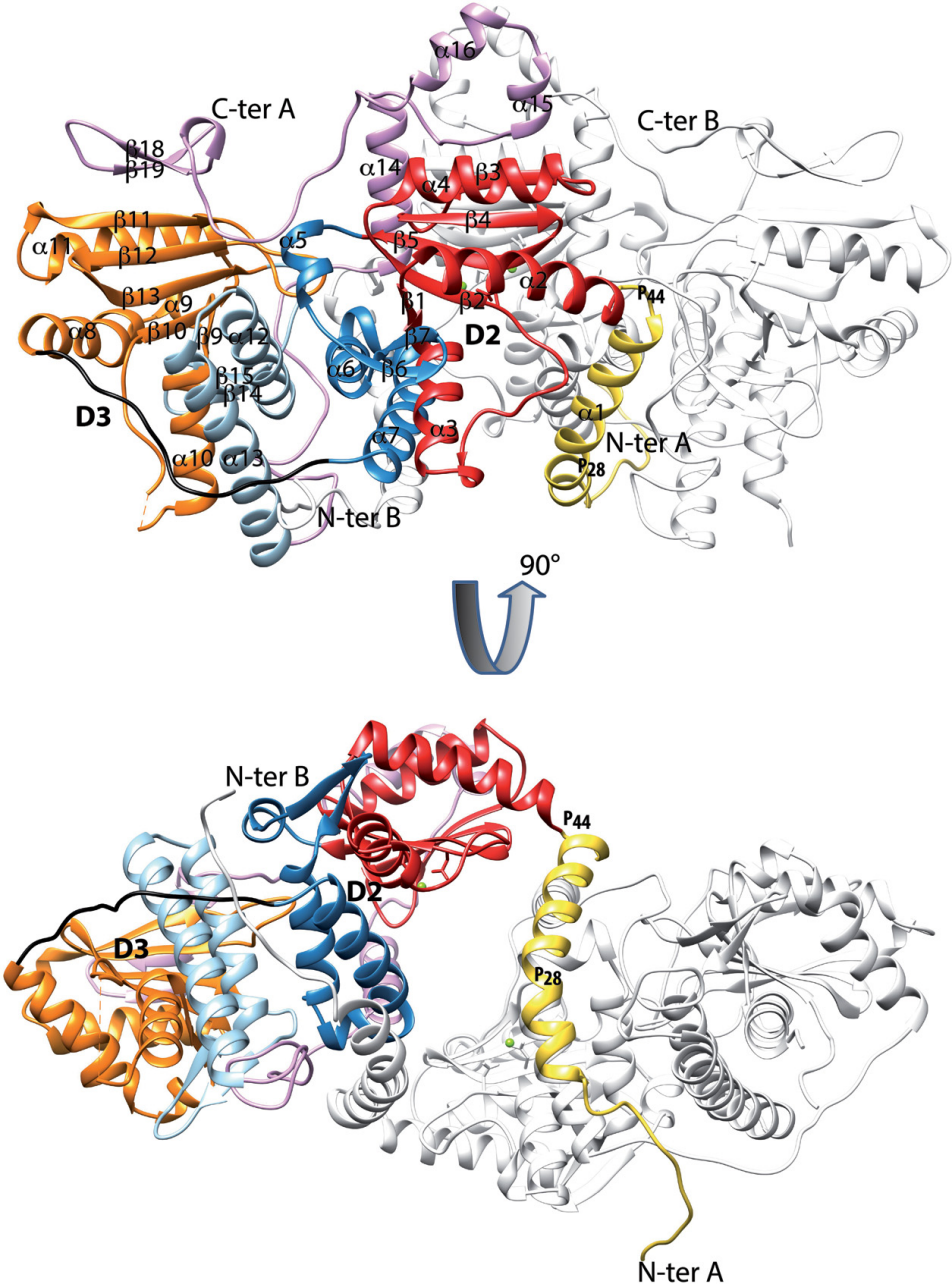
Structure of the polyA polymerase Mg561 of Megavirus chilensis. Ribbon representation of the Mg561 homodimer composed of the dimerization domain (D1), the catalytic domain (D2) including the canonical NT domain, the duplicated domain (D3) and the D4 domain. The 90° rotation highlights the central tunnel between the two monomers. Figure 1 color code is applied to one monomer to delineate the D1– D4 domains; the second monomer is in light gray. Cation locations are marked as green balls.

We determined the Mimivirus and M. chilensis PAP crystallographic 3D-structures in order to identify the determinants of their processivity. Since they shared the same characteristic features, we will refer to the highest resolution structure of Mg561 (Supplementary Table S1 and S2). Despite their lack of sequence similarity (15% of identical residues), the DALI search (50) returned the VP55 Vaccinia virus PAP (2GA9) as the best structural homologue of the Mg561 monomers (Z-score between 5 and 6, Cα-rmsd of 3.8 Å over 248 residues and Table S2). As predicted from the sequence comparison of the mimiviruses PAPs with other viral PAPs (Figure 1), their structures share four subdomains (Figure 3). The D5 domain unique to the Mimivirus PAP is disordered in the crystal structure.

#### The dimerization domain

**Figure 4.**
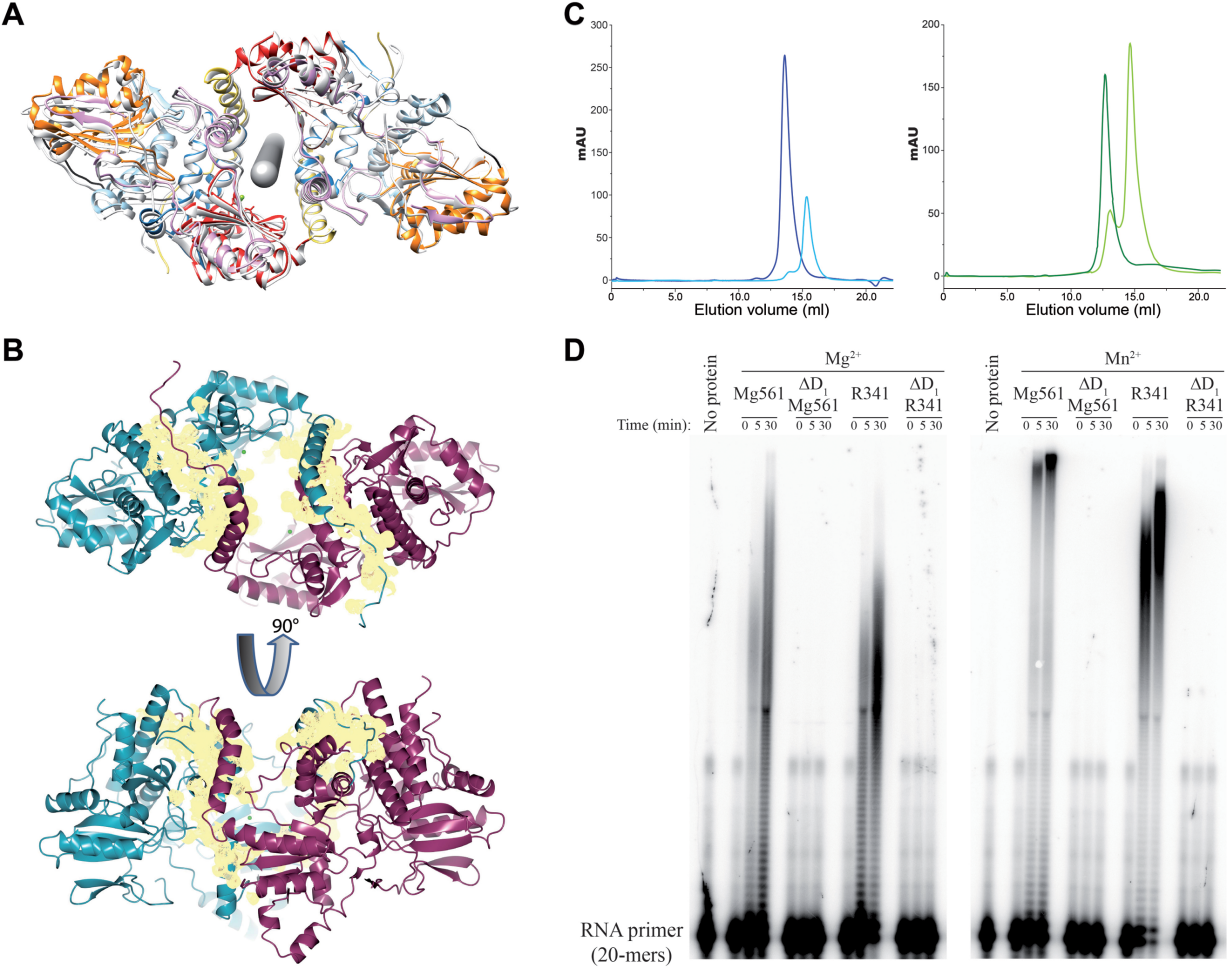
Molecular determinant of mimiviruses PAP dimerization and processivity. (**A**) Mg561 dimer symmetry. Rotational symmetry axis is shown in gray. Figure 1 color code is applied to one monomer and the second monomer is in light gray. (**B**) Buried surface area of the Mg561 dimer. The dimer interface buries a surface area (yellow) of about 3200 Å^2^, which corresponds to 11.5% of each monomer's solvent accessible surface area (about 27 500 Å^2^). The first monomer is in cyan, the second one is in magenta. Cation locations are marked as green balls. (**C**) Gel filtration analysis of ΔD1 H_6_c-Mg561 and ΔD1 H_6_c-R341 mutants. Overlay of UV traces of the full-length H_6_c-Mg561 (dark blue curve) and ΔD1 H_6_c-Mg561 (light blue curve) proteins (Left panel) and of the full-length H_6_c-R341 (dark green curve) and ΔD1 H_6_c-R341 (light green curve) proteins (Right panel). Full-length H_6_c-Mg561 and H_6_c-R341 eluted as a single peak corresponding to a dimeric state: apparent molecular masses were 116.8 and 176.7 kDa, respectively, *versus* the calculated ones, 125.7 and 136.8 kDa. At least 90% of the ΔD1 H_6_c-Mg561 and ΔD1 H_6_c-R341 protein eluted as monomers: apparent molecular masses were 57.8 and 71.1 kDa *versus* the calculated 57.7 and 63.4 kDa. GE Healthcare markers were used for calibration. (**D**) PolyA polymerase assay of H_6_c-Mg561, ΔD1 H_6_c-Mg561, H_6_c-R341 and ΔD1 H_6_c-R341 on a 20-mers RNA primer in the presence of Mg^2+^ or Mn^2+^ as catalytic ions. Time course is in min.

As observed in solution (see later), the Mg561 crystal structure reveals a very stable homodimer with an almost perfect two-fold symmetry axis (179°) and a 3200 Å^2^ buried surface area upon dimerization (Figures 3 and 4). The dimer structure highlights a large tunnel at the interface between the two monomers. The N-terminal domain (D1) encompasses an extended strand followed by a long helix (α_1_) bent at the P_28_ residue (Figures 1 and 3). In each monomer, this helix creates a domain swap, and makes contact with the D4 loop located between α_13_ and β_16_ and the α_7_ helix of the D2 domains of the other monomer to form the dimer structure. In the VP55 structure (PDB: 2GA9, (26)), the equivalent helix (α_8_, Figure 1B) is making contacts with the D4 (α_18_ and β_16_) and the D2 (α_15_) domains of the same molecule. In Mg561, next to the α_1_ helix, another proline residue (P_44_) induces a change in orientation of the main chain, making the first helix of the domain D2 (red) almost perpendicular to the α_1_ helix. We investigated if VP55 could form a related dimer at the high protein concentration found in crystals by scrutinizing the crystallographic structure of the unliganded monomeric VP55 containing two monomers per asymmetric unit (PDB: 3OWG, (51)). Even using the symmetry related molecules, no occurrences of such a dimer was visible. Reciprocally, none of the symmetry related molecules in the Mg561 and the R341 structure reproduce the dimer found in the VP55 structure. To further characterize the molecular determinants of the dimerization of Mg561 and the role of the dimerization on the polyadenylation activity, we made deletion of the Mg561 (ΔD1 Mg561) and R341 (ΔD1 R341) N-terminal domains. Gel filtration experiments revealed that the mutants were monomeric and although they still co-purified with RNA, they were inactive (Figure 4), suggesting that the dimeric state and the N-terminal domain were required for the polyadenylation activity.

#### The vPAPs catalytic domain

**Figure 5.**
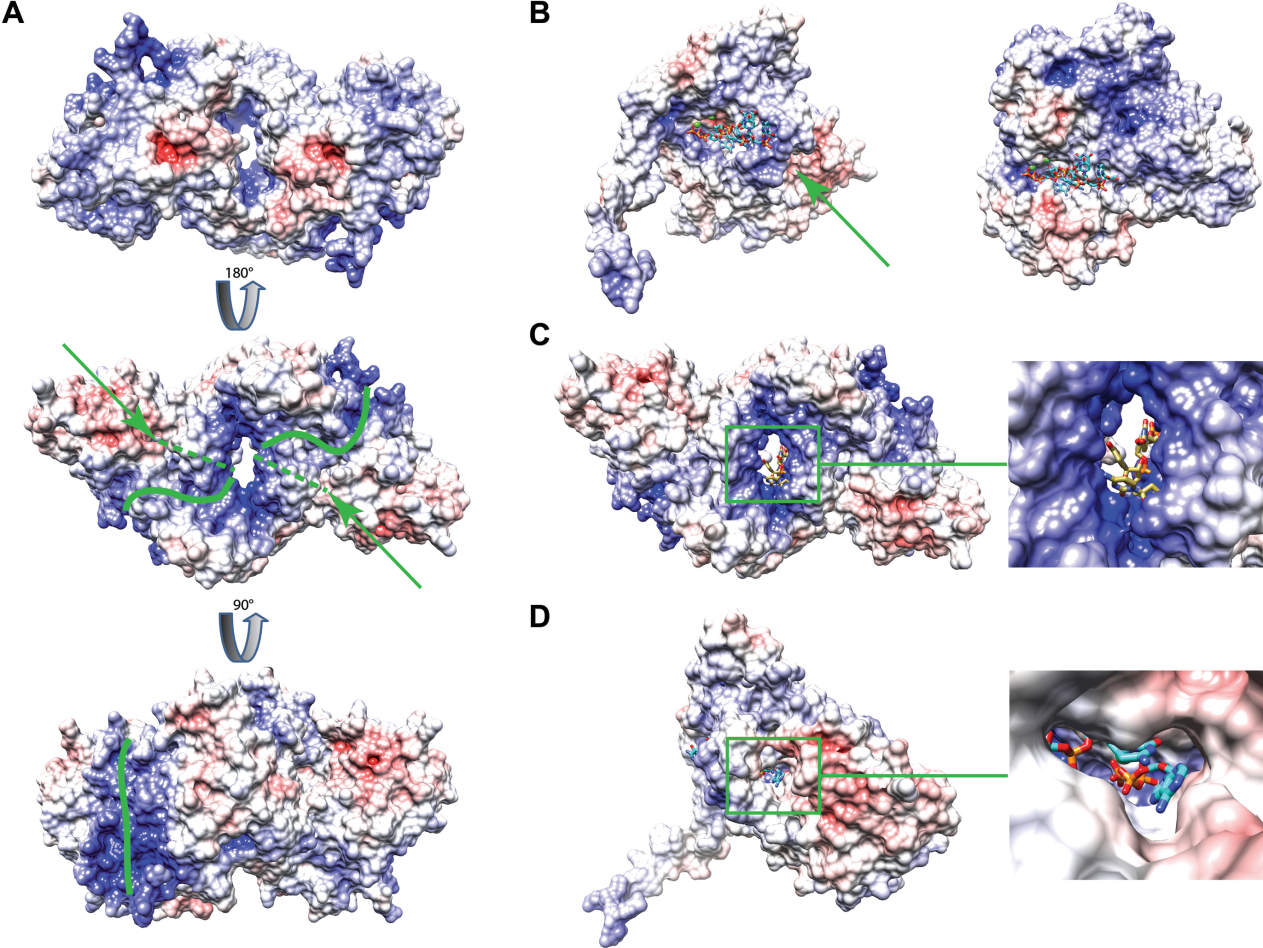
Electrostatic surface representation of the Mg561 protein. (**A**) Electrostatic surface of the Mg561 dimer. The green lines correspond to the possible RNA binding sites in the positively charged grooves (blue). The green arrows point the position of the second tunnel and the dashed lines delineate the tunnel path under the surface. Contour levels: red, V < −10 kcal/mol; white, −10 kcal/mol < V < +10 kcal/mol; blue, V > +10 kcal/mol. (**B**) Proposed mimiviruses PAP RNA and ATP binding mode (left panel, Mg561 monomer) based on the superimposition of the VP55 complex with ATP and RNA (PDB 3ERC) on Mg561 (right panel). Predicted cation sites are marked as green balls. (**C**) As in (B) but with the Mg561 dimer (left panel). Close-up of the central tunnel (right panel). (**D**) Proposed ATP entry route pointed by the green arrow (left panel). Close-up of the tunnel through the ATP binding site (right panel).

The second domain (D2) exhibits the typical topology of a nucleotidyltransferase (NT) domain made of a mixed five-stranded β sheet and two connecting helices and, as for the VP55 structure, the two helices pack against one side of the β sheet parallel to the β strands (Figures 1B and 3). The canonical acidic residues D_94_-E_96_-D_143_ are properly positioned and ordered in the crystal structure as evidenced by the 2Fo-Fc electron density maps contoured at 1σ on the of M. chilensis and Mimivirus PAP active sites (Supplementary Figure S6A). A water molecule replacing the Mg^2+^ ion usually found in structures in complex with ATP is used to help locate the active sites in the figures (Figures 3 and 5B, Supplementary Figure S4). The helix-turn motif within the β sheet presents a 10-residues longer loop relative to the Vaccinia virus PAP structure. This loop stabilizes the α_1_ helix of the same monomer in an orientation perpendicular to D2. This domain thus appears equivalent to the catalytic domain of VP55 made of an NT domain extended by one helix (Mg561 α_5_, VP55 α_12_), two antiparallel strands (β_6_-β_7_) and terminated by two parallel helices (Mg561 α_6_-α_7_, VP55 α_13_-α_15_). This topology thus defines the catalytic domain of viral PAPs (Figure 1B).

#### A duplication event at the origin of the internal symmetry of the vPAPs

The third domain (D3) of the Mg561 structure surprisingly mimics the catalytic domain topology (Figures 1B and 3) leading to an internal symmetry between the D2 and D3 domains (rotation of 162°, 2.4 Å translation and Cα-rmsd > 2.0 Å, Supplementary Figure S4, Table S2). Two of the three catalytic residues (F_286_, E_288_, K_342_) are replaced by non-canonical residues in D3, and an even longer insertion within the helix-turn motif (37 amino-acids) of the NT-like subdomain is present (Supplementary Figure S2). An equivalent of the α_5_ helix is also missing. Unexpectedly, the Vaccinia virus PAP D3 domain also follows this topology (Cα-rmsd >5 Å with Mg561 D2 and ∼2.5 Å with Mg561 D3, Supplementary Figure S4, Table S2) but with an extra β strand (β_8_) at the N-terminus of the NT-like subdomain replacing the canonical first helix (Figure 1B and Supplementary Figure S2). In the VP55 structure there is a rotation of 179° and a translation of 5 Å between the two subdomains leading to a Cα-rmsd of ∼2.6 Å (Supplementary Figure S4, Table S2). The equivalents of the α_10_ and α_12_ helices of the NT domain (α_3_ and α_5_ in Mg561) are also missing (Figure 1B). This suggests that despite the lack of sequence homology between the two domains, the D3 domain might have originated from an ancestral duplication of the catalytic domain, a duplication that might be a hallmark of the PAPs of all large DNA viruses. The two subdomains appear more divergent in the VP55 structure (Table S2), which could also explain why the internal symmetry of the molecule was not initially recognized.

#### The D4 domain

In Mg561, the D4 domain of monomer A extends at the interface between the two monomers, making contacts with the α_1_ helix of monomer B. It then runs toward the N-terminal end of monomer A, providing an extra β strand (β_17_) antiparallel to β_3_ of its D2 domain, instead of the two β strands provided by the D4 domain in the VP55 structure (β_17_ and β_18_). It finally runs back to terminate on top of the D3 domain of monomer A, the end of the structure being disordered (17 and 15 residues for A and B, respectively). The interaction with the D3 domain is stabilized by a conserved disulfide bridge (C_350_-C_426_) connecting the beginning of the β_14_ strand and the β_16_ strand in the D4 domain (Figure 1). The two D4 domains delineate what we defined as the entrance of the central tunnel at the interface of the dimer. This domain is the most basic in both sequences (predicted isoelectric points >10). It is worth noticing that the D5 extension unique to the Mimivirus PAP is disordered in the crystal structure and we thus have no clues on its molecular structure or function.

#### ATP binding site and nucleic acid recognition

Electrostatic potential calculations highlight intensely positively charged grooves on each side of the entrance of the central tunnel, which is contributed mostly by the positively charged D4 domains and to a lesser extent by the D2, D3 domains (Figure 5A and Supplementary Figures S5 and S6B). RNAs could therefore interact with the PAP through the positively charged grooves and reach the catalytic site through the 10 Å diameter wide central tunnel making the two catalytic sites accessible for a ssRNA molecule. We refer to this side as the entrance side. The enzyme processivity could be explained by the dimeric state of the PAP which positions and stabilizes the RNA molecule. The superimposition of the catalytic domains of Mg561 with the Vaccinia virus ATP-bound VP55 chain from the VP55-VP39 heterodimer structure (PDB 2GA9) showed that the residues involved in ATP binding were structurally conserved and ordered in the crystal structure as evidenced by the 2Fo-Fc electron density maps contoured at 1σ on the M. chilensis and Mimivirus active sites (Supplementary Figure S6A). As suggested by the electrostatic potential properties of the molecular surface, the superimposition of one monomer of the Mg561 structure with VP55 in complex with ATP and RNA (PDB 3ERC) seems to confirm that both the ATP and the RNA molecule could bind Mg561 in a similar way to VP55 (Figure 5B) and that the RNA molecule could enter through the central tunnel formed by the dimer (Figure 5C). We thus propose that the highly basic D4 domains assume the RNA binding function performed by VP39 in the VP55-VP39 heterodimer (Supplementary Figure S6B). We also observed that a second tunnel, visible on each side of the central tunnel, led directly to the ATP binding site and could therefore be the entry path for ATP toward the active site (Figure 5D). The processivity factor VP39 binds VP55 at this location and the tunnel is absent in the VP55 structures as well as in its complex with VP39 (2GA9 (26), 3OWG (51)). This major difference, where ATP could flow continuously through this tunnel, could also contribute to the processivity of the mimiviruses PAPs. Interestingly, positively charged residues are lining the central tunnel and are provided by α_1A_ (R_24_-K_32_-R_35_) and α_7B_ (R_189_-K_192_-R_196_) for one side of the tunnel and α_1B_ and α_7A_ for the other (Supplementary Figure S6C). These charged residues are mostly conserved in the mimiviruses sequences (Supplementary Figure S2). They could generate an outlet for the inorganic pyrophosphate (PPi) produced during the polymerization reaction, again contributing to the PAPs processivity.

### 3′-end hairpin recognition and cleavage

**Figure 6.**
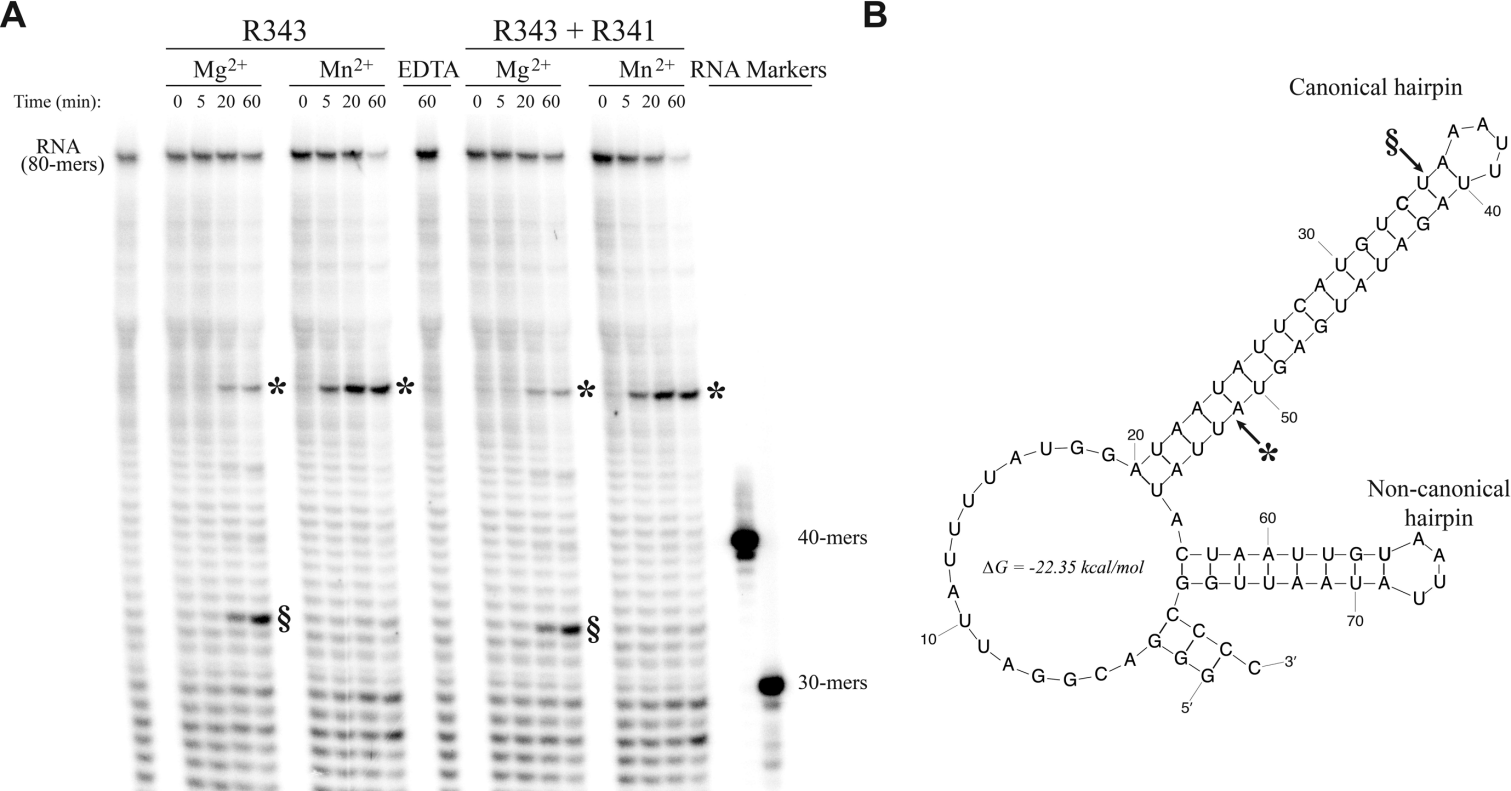
Hairpin recognition and processing. (**A**) RNase activity of the RNase III-like R343 or of a mixture of R343 and R341 on a 5′-^32^P-labeled short R418 RNA with Mg^2+^ or Mn^2+^. Asterisks and section signs mark the 51nt and the 34nt product, respectively. (**B**) Mfold secondary structure prediction (52) of the RNA used in (A). Cleavage sites are marked by arrows.

We synthesized *in vitro* the M. chilensis Mg592 transcript with its 3′UTR-hairpin as predicted by Mfold (52) and analyzed the polyadenylated products produced by the vPAPs (Supplementary Figure S7). All transcripts were polyadenylated at their 3′-extremities when using Mg561, R341, or the bacterial PAP used as a control, confirming that other proteins were required for the recognition and processing of the hairpin. The Mimivirus and M. chilensis PAPs belong to a conserved gene cluster predicted to also encode a protein with tandem RNAse III-like domains (R343 in Mimivirus and Mg559 in M. chilensis). Interestingly, the structure of the eukaryotic enzyme Rnt1p, which has a single RNase III domain, in complex with an RNA hairpin (PDB: 4OOG (53)) reveals that Rnt1p recognizes its hairpin substrate as a homodimer, allowing the excision of the hairpin structure. In the Mimivirus and M. chilensis RNAse III-like sequences, the residues involved in the dimer formation, as well as the ones responsible for the dsRNA binding, are conserved and both dsRNA binding domains have predicted isoelectric points > 10 as expected for nucleic acids binding domains. The catalytic as well as the metal binding residues are perfectly conserved in the N-terminal RNAse domain, while the residues involved in metal coordination are not conserved in the second RNAse domain. This suggested that the mimiviruses RNase III-like enzyme, as a momomer with two RNase III-like domains, can recognize the hairpin in the same way than the homodimeric Rnt1p, but is unable to excise it. Instead, it could perform a nick within the hairpin, allowing polyadenylation by the viral PAP inside the hairpin. To validate this hypothesis, we produced the Mimivirus R343 RNAse III-like protein and addressed its enzymatic activity *in vitro* on a RNA corresponding to the 3′-end of the of the Mimivirus R418 transcripts (Figure 6). Our results showed that R343 was able to cleave this RNA and generate two cleavage products inside the canonical hairpin in a metal-dependent manner. In the presence of Mg^2+^, a major product of 34nt and a minor one of 51nt were observed while only that of 51nt was observed in the presence of Mn^2+^. Interestingly, the major product observed in the presence of Mg^2+^ corresponded to the polyadenylation site previously identified *in vivo* for this transcript (22), suggesting that Mg^2+^ was preferentially used by R343 in cell. More importantly, this demonstrated that R343 is the enzyme responsible for the canonical hairpin recognition and its specific cleavage (Supplementary Figure S1C, step 5). The presence of the R341 PAP had no impact on the cleavage. To elucidate if R343 and R341 were sufficient for 3′-end mRNA maturation we incubated sequentially the two enzymes with the Mg592 transcripts and analyzed the polyadenylated products. We also sequenced the corresponding *in vivo* transcripts extracted from *A. castellanii* cells infected by M. chilensis. We observed the same polyadenylation site within the hairpin after R343 incubation with Mg^2+^ or Mn^2+^ but this site was different from that of the transcripts polyadenylated *in vivo* (Supplementary Figure S7). This suggested that a third protein could be required to direct the *in vivo* location of the nick even if we cannot rule out the fact that the Mimivirus and the M. chilensis enzymes could cleave at different sites on the same hairpin and thus the Mg559 protein could produce the same nick *in vivo* and *in vitro* on the Mg592 hairpin.

### Mg18 is a SAM-dependent ribose 2′O MTase

**Figure 7.**
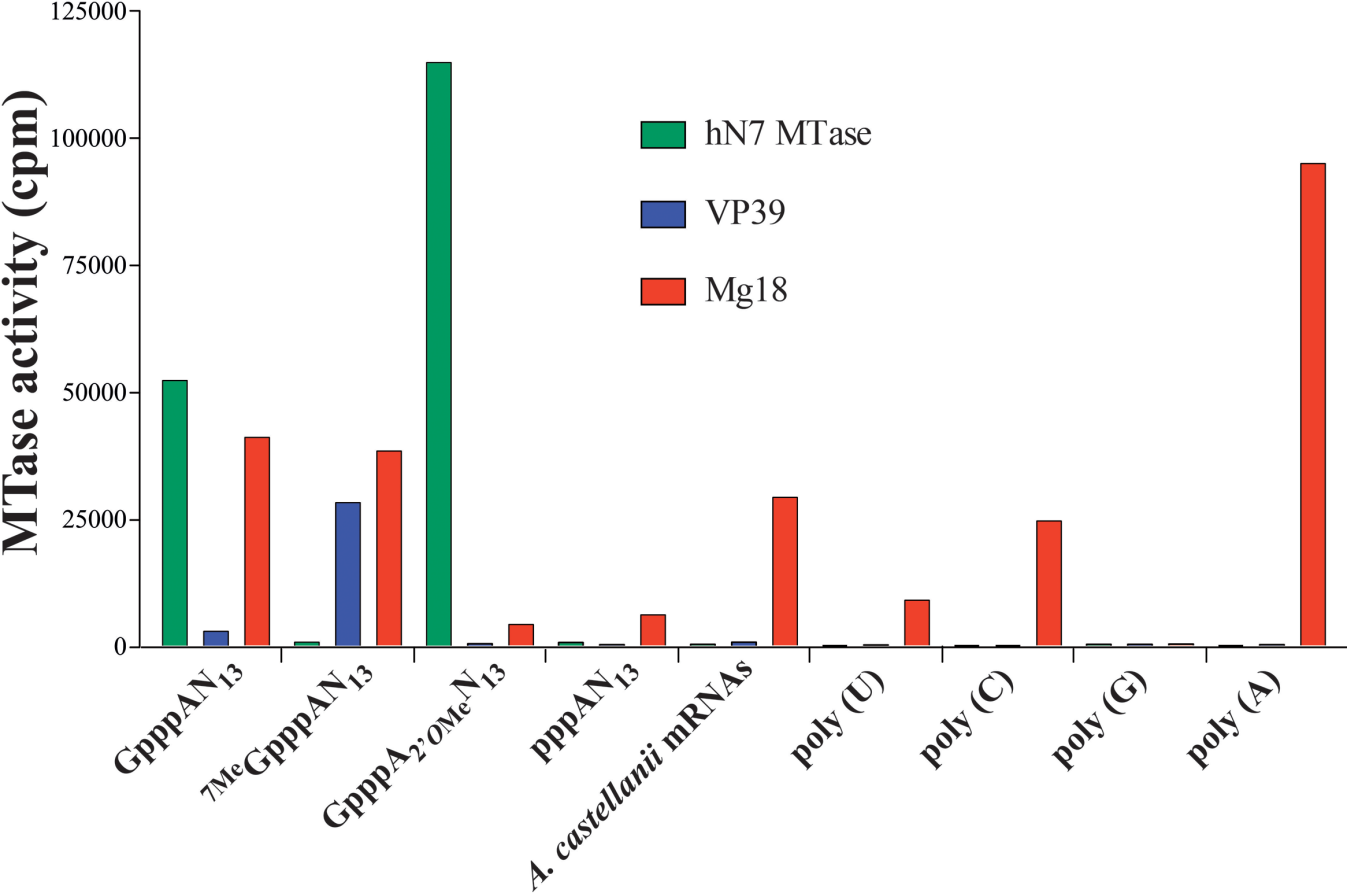
Mg18 2′O MTase activity. AdoMet-dependent MTase assays were performed on equimolar amounts of short capped RNAs substrates (GpppAN_13_), N7- or 2′O-methylated capped RNA substrates (^7Me^GpppAN_13_ or GpppA_2′OMe_N_13_), *A. castellanii* mRNAs or homopolymeric poly (U), (C), (G) and (A). Human N7 MTase and Vaccinia virus VP39 were use as controls.

Even though we postulated that a cap specific 2′O MTase is present in the conserved gene cluster encoding the mimiviruses mRNA capping enzyme, we wondered whether the Mg18, like its Vaccinia virus VP39 homologue, could retain the 2′O MTase activity (Supplementary Figure S1C, step 3). Sequence analysis revealed that Mg18 harbors the conserved catalytic K-D-K-E tetrad shared by all known 2′O MTases including that of poxvirus VP39 (33), flavivirus (54), coronavirus (55) and rhabdovirus (56). In the VP39 structure cap recognition is made by the stacking of two aromatic side chains (Y_22_ and a F_180_) and hydrogen bonding with two acidic side chains (D_182_, E_233_). The first aromatic residue is conserved in the Mg18 sequence (Y_59_) while the loop bearing the F_180_ and the D_182_ residues is 4 amino acids shorter in the Mg18 sequence suggesting that Mg18 may not be as specific for cap residues as VP39 (57). We compared the MTase activities of Mg18, VP39 and the Human N7 MTase (hN7 MTase) on several RNA substrates (Figure 7). First, Mg18 has a much greater activity on the capped GpppAN_13_ than the uncapped pppAN_13_, therefore is dependent upon 5′-guanylylation. In contrast to VP39, Mg18 had comparable activity with ^7Me^GpppAN_13_ and GpppAN_13_ and therefore, its activity does not dependent on the cap methylation status. Mg18 is about 10 times less efficient on 2′O-methylated capped RNAs (GpppA_2′*O*Me_N_13_) than on N7-methylated or unmethylated caps (^7Me^GpppAN_13_ or GpppAN_13_), indicating that the methylated residue in GpppA_2′*O*Me_N_13_ is the methylation target. As expected, the addition of Mg561 had no influence on the 5′-GpppA specific Mg18 MTase activity (Supplementary Figure S8). Then we investigated the methylation state of the *A. castellanii* polyA+ mRNA caps using VP39 and the hN7 MTase. The *A. castellanii* mRNAs were not methylated by the two MTases, suggesting that their caps were already methylated both at the N7 and at the ribose 2′O positions. To our surprise, Mg18 exhibited a strong MTase activity on the polyadenylated *A. castellanii* mRNAs (Figure 7), suggesting that Mg18 had a second distinct activity: the expected 5′-GpppA specific 2′O MTase activity, and another cap independent one. To investigate this additional function of Mg18, we assayed Mg18 activity on uncapped homopolymeric poly (A), (U), (C) and (G) substrates. We found an even stronger MTase activity of Mg18 on polyadenylates. In the absence of any cap structure, Mg18 is able to methylate the ribose 2′O position of internal nucleotides with a clear specificity for adenylates suggesting it can also methylate the mRNAs polyA tails (Supplementary Figure S1C, step 7).

## DISCUSSION

Transcripts maturation is a central step in the process of eukaryotic gene expression and involves complex protein machineries responsible for transcription termination, 5′-end capping of the mRNA, N7 and/or 2′O methylation of the cap, mRNA 3′-end cleavage and polyadenylation. The maturation enhances the translation by increasing the mRNAs stability and their affinity for the ribosome. Viruses infecting eukaryotes have evolved their own strategies to favor their genes expression either by hijacking the host machinery or using virally encoded proteins. For the members of the mimiviruses sub-family, the transcription enzymes are virally encoded and loaded in the virions, allowing transcription to proceed immediately upon infection. They all share a specific signal governing their transcripts polyadenylation, which correlates with a hairpin structure. Mimivirus and M. chilensis share a remote homologue of viral and cellular PAP located in a cluster of three conserved genes transcribed as a polycistronic mRNA encompassing the three protein coding regions (22).

We verified that the Mimivirus and M. chilensis predicted PAPs were able to perform polyadenylation. These proteins were not only active, but also intrinsically self-processive, generating >700 nucleotides long polyA tails. To elucidate the molecular basis of this processivity we determined the Mimivirus and M. chilensis PAP crystallographic 3D structures, which revealed two topologically identical subdomains with a nucleotidyltransferase fold. A similar topology is still recognizable in the Vaccinia virus PAP structure and appears conserved in other NCLDVs, suggesting that an ancestral duplication was at the origin of the vPAPs. Surprisingly, the Mimivirus and M. chilensis enzymes are homodimeric and their 3D-structures suggested that the dimer stability depended on the N-terminal domain, the deletion of which lead to monomeric and enzymatically inactive proteins although still able to bind RNA. Thus, while other PAPs form heterodimers with processivity factors, the Mimivirus and M. chilensis PAP become processive upon homodimerization. Since the Mimivirus and M. chilensis PAP add polyA tails at the 3′-end of any mRNA, additional proteins are required to recognize the hairpin structure and cleave it prior polyadenylation. We showed that this role is played by the product of the third gene of the PAP gene cluster, which is able both to recognize and cleave the mRNA, within the hairpin. However, the RNase III-like sequences are lacking the elements acting as a ruler in eukaryotic enzymes (53), defining the precise location of the hairpin cleavage. The R343 enzyme cleavage site *in vitro* is not the same as *in vivo* suggesting that another factor is required to achieve the highly specific 3′-end processing of viral transcripts. We hypothesize that the second gene of the cluster (R342 and Mg560) encoding a protein made of a N-terminal basic domain and a C-terminal acidic one, could play the role of the ruler through its specific interaction with the RNAse and the RNA. This would need to be addressed experimentally.

Another step of mRNA maturation corresponds to the mRNA capping and its methylation. All members of the mimiviruses sub-family encode a mRNA capping enzyme also performing the N7 methylation of the cap (31) and a cap-specific guanine-N2 methyltransferase, forming a 2,7-dimethylguanosine DMG cap which could favor viral protein synthesis (32). The mRNA capping enzyme genes are located next to genes predicted to encode a 2′O methyltransferase and both proteins are loaded in the virions. Homologs of that predicted 2′O MTase are found in all NCLDVs, including CroV (CroV442) -where it is also loaded in the virion (58)-, PgV (9) and OLPV (10). A 2′O MTase activity was also reported in the virions of Asfarviruses (16). Therefore, both proteins from the mRNA capping cluster could be involved in the mRNA 5′-end maturation. In contrast, the poxviruses exhibit an inactive homologue of this 2′O MTase (D12) the function of which is performed by the dual function VP39 protein (59,60). M. chilensis encodes a homologue of the Vaccinia VP39 factor, the Mg18 protein, which retained the 2′O MTase activity and is also able to internally methylate the mRNAs polyA tails (Supplementary Figure S1C, step 7). The polyA tail synthesis and its internal methylation could thus be performed concomitantly through the interaction between M. chilensis PAP and Mg18. To date, the Flaviviruses NS5 MTase is the only enzyme reported to specifically methylate the internal 2′O adenosines of the viral genomic RNA to increase its stability (61). M. chilensis and the other Mimiviridae able to internally methylate their mRNAs could take advantage of this property. Compared to the host mRNA, the viral mRNA longer polyA tails might allow the viruses to further increase their stability by delaying their degradation in the cytoplasm and thus favor their translation over the host's mRNAs. Since the natural host of M. chilensis is not known, the differences in transcripts maturation observed between Mimivirus and M. chilensis could be the signature of their specificity toward their natural hosts.

The use of a hairpin signal for viral mRNA maturation has been reported for the first time in Mimivirus and is universally used by all mimiviruses (18,22). Their genomes are AT-rich which could explain why they use a structural signal for polyadenylation, instead of the ‘AATAAA’ canonical sequence which can be randomly found all along the genomes. Some other AT-rich DNA viruses such as the poxviruses present conserved polyuridylate sequences at specific positions with respect to the genes 3′ ends, which are used as termination signals. As a result, there is a very large number of distinct polyadenylated 3′-ends consistent with the absence of a highly specific recognition motif (62). In Mimivirus, the few occurrences where the AATAAA canonical signal is used, its location relative to the transcription end is also much less stringent than for the palindrome structure. Hairpin structures-based processes have been documented in the cellular world for RNA maturation. In the prokaryotic Rho-independent transcription termination process (63), the transcription stops when the newly synthesized RNA molecule forms a short G-C-rich hairpin followed by a run of polyuridylates, due to the release of the RNA from the DNA template. In eukaryotes, the histones mRNA maturation also involves short hairpin structures but they are not polyadenylated and the hairpin is formed by endonucleolytic cleavage of the pre-mRNA (64). Non-coding RNA 3′-end maturation also involves hairpin structures (small nuclear RNA, small nucleolar RNA, miRNA in eukaryotes, rRNA in prokaryotes) (65). It is a dimeric RNAse III, which is involved in the hairpin recognition and performs the hairpin excision but the resulting products are not polyadenylated.

The alternative solutions evolved by mimiviruses to perform transcription, a central cellular process, questions the general idea that viruses are merely hijacking the host cell machinery or recruiting cellular processes through lateral gene transfer. Even if some viral enzymes are reminiscent of cellular ones, the mimiviruses overall machinery is markedly different. Overall, the viral machinery appears less complex than the multiprotein machinery needed for cellular RNA maturation. A single protein is required to both cap the mRNA and methylate them in N7. The 3′-end maturation of transcripts also involves a minimal machinery made of two proteins instead of the five required in eukaryotes. The newly discovered Pithovirus sibericum, a 30 000 years old virus revived from permafrost, although not a member of the Mimiviridae, also possesses an AT-rich genome and obeys the hairpin rule for its transcripts maturation (66). Interestingly, it encodes a RNAse III-like protein suggesting it uses a similar transcripts maturation process.

By definition, viruses are forced to rely on the host translation apparatus. This creates a fundamental bottleneck for their mRNAs to gain access to protein synthesis, in particular for those exclusively replicating in the cytoplasm. To win this showdown against the cell own mRNAs, viruses must constantly evolve new strategies. Different virus families have independently raised different solutions ranging from ribosomes editing, cellular mRNAs degradation, or stabilization of their own messengers (for review see (67)). The minimum molecular machinery set up by the Mimiviridae to produce and stabilize their own mRNA is another illustration of these variations on a theme in the arms-race contest with their host.

## ACCESSION NUMBERS

The 3D structures have been deposited in the Protein Data Bank under the accession numbers: 4P37 (Mg561) and 4WSE (R341).

## SUPPLEMENTARY DATA

Supplementary Data are available at NAR Online.

SUPPLEMENTARY DATA

## References

[1] Raoult D., Audic S., Robert C., Abergel C., Renesto P., Ogata H., La Scola B., Suzan M., Claverie J.M. (2004). The 1.2-megabase genome sequence of Mimivirus. Science.

[2] Colson P., Yutin N., Shabalina S.A., Robert C., Fournous G., La Scola B., Raoult D., Koonin E.V. (2011). Viruses with more than 1,000 genes: Mamavirus, a new Acanthamoeba polyphaga mimivirus strain, and reannotation of Mimivirus genes. Genome Biol. Evol..

[3] Desnues C., La Scola B., Yutin N., Fournous G., Robert C., Azza S., Jardot P., Monteil S., Campocasso A., Koonin E.V. (2012). Provirophages and transpovirons as the diverse mobilome of giant viruses. Proc. Natl. Acad. Sci. U.S.A..

[4] Yoosuf N., Yutin N., Colson P., Shabalina S.A., Pagnier I., Robert C., Azza S., Klose T., Wong J., Rossmann M.G. (2012). Related giant viruses in distant locations and different habitats: Acanthamoeba polyphaga moumouvirus represents a third lineage of the Mimiviridae that is close to the megavirus lineage. Genome Biol. Evol..

[5] Yoosuf N., Pagnier I., Fournous G., Robert C., Raoult D., La Scola B., Colson P. (2014). Draft genome sequences of Terra1 and Terra2 viruses, new members of the family Mimiviridae isolated from soil. Virology.

[6] Arslan D., Legendre M., Seltzer V., Abergel C., Claverie J.M. (2011). Distant Mimivirus relative with a larger genome highlights the fundamental features of Megaviridae. Proc. Natl. Acad. Sci. U.S.A..

[7] Saadi H., Pagnier I., Colson P., Cherif J.K., Beji M., Boughalmi M., Azza S., Armstrong N., Robert C., Fournous G. (2013). First isolation of Mimivirus in a patient with pneumonia. Clin. Infect. Dis..

[8] Fischer M.G., Allen M.J., Wilson W.H., Suttle C.A. (2010). Giant virus with a remarkable complement of genes infects marine zooplankton. Proc. Natl. Acad. Sci. U.S.A..

[9] Santini S., Jeudy S., Bartoli J., Poirot O., Lescot M., Abergel C., Barbe V., Wommack K.E., Noordeloos A.A., Brussaard C.P. (2013). Genome of Phaeocystis globosa virus PgV-16T highlights the common ancestry of the largest known DNA viruses infecting eukaryotes. Proc. Natl. Acad. Sci. U.S.A..

[10] Yau S., Lauro F.M., DeMaere M.Z., Brown M.V., Thomas T., Raftery M.J., Andrews-Pfannkoch C., Lewis M., Hoffman J.M., Gibson J.A. (2011). Virophage control of antarctic algal host-virus dynamics. Proc. Natl. Acad. Sci. U.S.A..

[11] Iyer L.M., Aravind L., Koonin E.V. (2001). Common origin of four diverse families of large eukaryotic DNA viruses. J. Virol..

[12] Kuznar J., Salas M.L., Vinuela E. (1980). DNA-dependent RNA polymerase in African swine fever virus. Virology.

[13] Minnigan H., Moyer R.W. (1985). Intracellular location of rabbit poxvirus nucleic acid within infected cells as determined by in situ hybridization. J. Virol..

[14] Claverie J.M., Abergel C., Ogata H. (2009). Mimivirus. Curr. Topics Microbiol. Immunol..

[15] Mutsafi Y., Zauberman N., Sabanay I., Minsky A. (2010). Vaccinia-like cytoplasmic replication of the giant Mimivirus. Proc. Natl. Acad. Sci. U.S.A..

[16] Salas M.L., Kuznar J., Vinuela E. (1981). Polyadenylation, methylation, and capping of the RNA synthesized in vitro by African swine fever virus. Virology.

[17] Renesto P., Abergel C., Decloquement P., Moinier D., Azza S., Ogata H., Fourquet P., Gorvel J.P., Claverie J.M. (2006). Mimivirus giant particles incorporate a large fraction of anonymous and unique gene products. J. Virol..

[18] Legendre M., Audic S., Poirot O., Hingamp P., Seltzer V., Byrne D., Lartigue A., Lescot M., Bernadac A., Poulain J. (2010). mRNA deep sequencing reveals 75 new genes and a complex transcriptional landscape in Mimivirus. Genome Res..

[19] Broyles S.S. (2003). Vaccinia virus transcription. J. Gen. Virol..

[20] Baroudy B.M., Moss B. (1980). Purification and characterization of a DNA-dependent RNA polymerase from vaccinia virions. J. Biol. Chem..

[21] Suhre K., Audic S., Claverie J.M. (2005). Mimivirus gene promoters exhibit an unprecedented conservation among all eukaryotes. Proc. Natl. Acad. Sci. U.S.A..

[22] Byrne D., Grzela R., Lartigue A., Audic S., Chenivesse S., Encinas S., Claverie J.M., Abergel C. (2009). The polyadenylation site of Mimivirus transcripts obeys a stringent ‘hairpin rule’. Genome Res..

[23] Yang Q., Nausch L.W., Martin G., Keller W., Doublie S. (2014). Crystal structure of human poly(A) polymerase gamma reveals a conserved catalytic core for canonical poly(A) polymerases. J. Mol. Biol..

[24] Balbo P.B., Bohm A. (2007). Mechanism of poly(A) polymerase: structure of the enzyme-MgATP-RNA ternary complex and kinetic analysis. Structure.

[25] Bai Y., Srivastava S.K., Chang J.H., Manley J.L., Tong L. (2011). Structural basis for dimerization and activity of human PAPD1, a noncanonical poly(A) polymerase. Mol. Cell.

[26] Moure C.M., Bowman B.R., Gershon P.D., Quiocho F.A. (2006). Crystal structures of the vaccinia virus polyadenylate polymerase heterodimer: insights into ATP selectivity and processivity. Mol. Cell.

[27] Rodriguez J.M., Yanez R.J., Rodriguez J.F., Vinuela E., Salas M.L. (1993). The DNA polymerase-encoding gene of African swine fever virus: sequence and transcriptional analysis. Gene.

[28] Condit R.C., Niles E.G. (2002). Regulation of viral transcription elongation and termination during vaccinia virus infection. Biochim. Biophys. Acta.

[29] Gershon P.D., Moss B. (1993). Uridylate-containing RNA sequences determine specificity for binding and polyadenylation by the catalytic subunit of vaccinia virus poly(A) polymerase. EMBO J..

[30] Gershon P.D., Moss B. (1993). Stimulation of poly(A) tail elongation by the VP39 subunit of the vaccinia virus-encoded poly(A) polymerase. J. Biol. Chem..

[31] Benarroch D., Smith P., Shuman S. (2008). Characterization of a trifunctional mimivirus mRNA capping enzyme and crystal structure of the RNA triphosphatase domain. Structure.

[32] Benarroch D., Qiu Z.R., Schwer B., Shuman S. (2009). Characterization of a mimivirus RNA cap guanine-N2 methyltransferase. RNA.

[33] Hodel A.E., Gershon P.D., Shi X., Quiocho F.A. (1996). The 1.85 A structure of vaccinia protein VP39: a bifunctional enzyme that participates in the modification of both mRNA ends. Cell.

[34] Lartigue A., Jeudy S., Bertaux L., Abergel C. (2013). Preliminary crystallographic analysis of a polyadenylate synthase from Megavirus. Acta Crystallogr. F.

[35] Peyrane F., Selisko B., Decroly E., Vasseur J.J., Benarroch D., Canard B., Alvarez K. (2007). High-yield production of short GpppA- and 7MeGpppA-capped RNAs and HPLC-monitoring of methyltransfer reactions at the guanine-N7 and adenosine-2′O positions. Nucleic Acids Res..

[36] Bricogne G., Vonrhein C., Flensburg C., Schiltz M., Paciorek W. (2003). Generation, representation and flow of phase information in structure determination: recent developments in and around SHARP 2.0. Acta Crystallogr. D Biol. Crystallogr..

[37] Blanc E., Roversi P., Vonrhein C., Flensburg C., Lea S.M., Bricogne G. (2004). Refinement of severely incomplete structures with maximum likelihood in BUSTER-TNT. Acta Crystallogr. D Biol. Crystallogr..

[38] Abergel C. (2004). Spectacular improvement of X-ray diffraction through fast desiccation of protein crystals. Acta Crystallogr. D Biol. Crystallogr..

[39] Kabsch W. (2010). Xds. Acta Crystallogr. D Biol. Crystallogr..

[40] McCoy A.J., Grosse-Kunstleve R.W., Adams P.D., Winn M.D., Storoni L.C., Read R.J. (2007). Phaser crystallographic software. J. Appl. Crystallogr..

[41] Krissinel E., Henrick K. (2007). Inference of macromolecular assemblies from crystalline state. J. Mol. Biol..

[42] Baker N.A., Sept D., Joseph S., Holst M.J., McCammon J.A. (2001). Electrostatics of nanosystems: application to microtubules and the ribosome. Proc. Natl. Acad. Sci. U.S.A..

[43] Jeudy S., Claverie J.M., Abergel C. (2006). The nucleoside diphosphate kinase from mimivirus: a peculiar affinity for deoxypyrimidine nucleotides. J. Bioenerg. Biomembr..

[44] Barral K., Sallamand C., Petzold C., Coutard B., Collet A., Thillier Y., Zimmermann J., Vasseur J.J., Canard B., Rohayem J. (2013). Development of specific dengue virus 2′-O- and N7-methyltransferase assays for antiviral drug screening. Antiviral Res..

[45] Altschul S.F., Madden T.L., Schaffer A.A., Zhang J., Zhang Z., Miller W., Lipman D.J. (1997). Gapped BLAST and PSI-BLAST: a new generation of protein database search programs. Nucleic Acids Res..

[46] Aravind L., Koonin E.V. (1999). DNA polymerase beta-like nucleotidyltransferase superfamily: identification of three new families, classification and evolutionary history. Nucleic Acids Res..

[47] Legendre M., Arslan D., Abergel C., Claverie J.M. (2012). Genomics of Megavirus and the elusive fourth domain of Life. Commun. Integr. Biol..

[48] Gershon P.D., Moss B. (1992). Transition from rapid processive to slow nonprocessive polyadenylation by vaccinia virus poly(A) polymerase catalytic subunit is regulated by the net length of the poly(A) tail. Genes Dev..

[49] Martin G., Keller W. (2007). RNA-specific ribonucleotidyl transferases. RNA.

[50] Holm L., Rosenstrom P. (2010). Dali server: conservation mapping in 3D. Nucleic Acids Res..

[51] Li H., Li C., Zhou S., Poulos T.L., Gershon P.D. (2013). Domain-level rocking motion within a polymerase that translocates on single-stranded nucleic acid. Acta Crystallogr. D Biol. Crystallogr..

[52] Zuker M. (2003). Mfold web server for nucleic acid folding and hybridization prediction. Nucleic Acids Res..

[53] Liang Y.H., Lavoie M., Comeau M.A., Abou Elela S., Ji X. (2014). Structure of a eukaryotic RNase III postcleavage complex reveals a double-ruler mechanism for substrate selection. Mol. Cell.

[54] Egloff M.P., Benarroch D., Selisko B., Romette J.L., Canard B. (2002). An RNA cap (nucleoside-2′-O-)-methyltransferase in the flavivirus RNA polymerase NS5: crystal structure and functional characterization. EMBO J..

[55] Bouvet M., Debarnot C., Imbert I., Selisko B., Snijder E.J., Canard B., Decroly E. (2010). In vitro reconstitution of SARS-coronavirus mRNA cap methylation. PLoS Pathog..

[56] Li J., Fontaine-Rodriguez E.C., Whelan S.P. (2005). Amino acid residues within conserved domain VI of the vesicular stomatitis virus large polymerase protein essential for mRNA cap methyltransferase activity. J. Virol..

[57] Hodel A.E., Gershon P.D., Quiocho F.A. (1998). Structural basis for sequence-nonspecific recognition of 5′-capped mRNA by a cap-modifying enzyme. Mol. Cell.

[58] Fischer M.G., Kelly I., Foster L.J., Suttle C.A. (2014). The virion of Cafeteria roenbergensis virus (CroV) contains a complex suite of proteins for transcription and DNA repair.

[59] Schnierle B.S., Gershon P.D., Moss B. (1992). Cap-specific mRNA (nucleoside-O2′-)-methyltransferase and poly(A) polymerase stimulatory activities of vaccinia virus are mediated by a single protein. Proc. Natl. Acad. Sci. U.S.A..

[60] Kyrieleis O.J., Chang J., de la Pena M., Shuman S., Cusack S. (2014). Crystal structure of vaccinia virus mRNA capping enzyme provides insights into the mechanism and evolution of the capping apparatus. Structure.

[61] Dong H., Chang D.C., Hua M.H., Lim S.P., Chionh Y.H., Hia F., Lee Y.H., Kukkaro P., Lok S.M., Dedon P.C. (2012). 2′-O methylation of internal adenosine by flavivirus NS5 methyltransferase. PLoS Pathog..

[62] Yang Z., Bruno D.P., Martens C.A., Porcella S.F., Moss B. (2011). Genome-wide analysis of the 5′ and 3′ ends of vaccinia virus early mRNAs delineates regulatory sequences of annotated and anomalous transcripts. J. Virol..

[63] Wilson K.S., von Hippel P.H. (1995). Transcription termination at intrinsic terminators: the role of the RNA hairpin. Proc. Natl. Acad. Sci. U.S.A..

[64] Marzluff W.F., Wagner E.J., Duronio R.J. (2008). Metabolism and regulation of canonical histone mRNAs: life without a poly(A) tail. Nat. Rev. Genet..

[65] Peart N., Sataluri A., Baillat D., Wagner E.J. (2013). Non-mRNA 3′ end formation: how the other half lives. Wiley Interdiscip. Rev. RNA.

[66] Legendre M., Bartoli J., Shmakova L., Jeudy S., Labadie K., Adrait A., Lescot M., Poirot O., Bertaux L., Bruley C. (2014). Thirty-thousand-year-old distant relative of giant icosahedral DNA viruses with a pandoravirus morphology. Proc. Natl. Acad. Sci. U.S.A..

[67] Walsh D., Mohr I. (2011). Viral subversion of the host protein synthesis machinery. Nat. Rev. Microbiol..

