# Supplementary material for: mRNA maturation in giant viruses: variation on a theme
Source: Nucleic Acids Res. 2015 Mar 16;43(7):3776–88. doi: 10.1093/nar/gkv224 (PMC4402537; doi:10.1093/nar/gkv224)
Supplement: SUPPLEMENTARY DATA [file supp_43_7_3776__index.html]

mRNA maturation in giant viruses: variation on a theme — mRNA maturation in giant viruses: variation on a theme — SUPPLEMENTARY DATA 

# mRNA maturation in giant viruses: variation on a theme

## SUPPLEMENTARY DATA

**Files in this Data Supplement:**

- SUPPLEMENTARY DATA
