# Supplementary material for: mRNA maturation in giant viruses: variation on a theme
Source: Nucleic Acids Res. 2015 Mar 16;43(7):3776–88. doi: 10.1093/nar/gkv224 (PMC4402537; doi:10.1093/nar/gkv224)
Supplement: SUPPLEMENTARY DATA [file supp_gkv224_nar-03565-a-2014-File009.docx]

**Supplementary data**

**mRNA maturation in giant viruses: variation on a theme**

Stéphane Priet^2^ †, Audrey Lartigue^1^ †, Françoise Debart^4^, Jean-Michel Claverie^1,3*^, Chantal Abergel^1*^

^1^ Structural and Genomic Information Laboratory, UMR 7256 (IMM FR 3479) CNRS Aix-Marseille Université, 163 Avenue de Luminy, Case 934, 13288 Marseille cedex 9, France.

^2^ Architecture et Fonction des Macromolécules Biologiques, CNRS UMR 7257, Aix-Marseille Université, 163 Avenue de Luminy, Case 932, 13288 Marseille cedex 9, France.

^3^ APHM, FR-13385 Marseille, France.

^4^ IBMM, UMR 5247, CNRS-UM1-UM2, Université Montpellier 2, Place Eugène Bataillon, 34095 Montpellier, France.

* To whom correspondence should be addressed. Tel: +33 4 91825422; Fax: +33 4 9182542; Email: Chantal.Abergel@igs.cnrs-mrs.fr, Jean-Michel.Claverie@igs.cnrs-mrs.fr

† The authors wish it to be known that, in their opinion, the first two authors should be regarded as joint First Authors

**Supplementary Table S1**

**Supplementary Table S2**

**Supplementary Figure S1**

**Supplementary Figure S2**

**Supplementary Figure S3**

**Supplementary Figure S4**

**Supplementary Figure S5**

**Supplementary Figure S6**

**Supplementary Figure S7**

**Supplementary Figure S8**

**Supplementary Table 1.** **Data collection and refinement statistics**

| **Crystal Parameters** | Mg561 | R341 |
| --- | --- | --- |
| Space group | *P*2_1_2_1_2_1_ | *C*2_1_ |
| Cell dimensions |  |  |
| *a*, *b*, *c* (Å) | 86.6, 96.1, 153.9 | 200.0, 69.6, 97.5 |
| α,β, γ (°) | 90.0, 90.0, 90.0 | 90.0, 105.7, 90.00 |
| Cell content | 2 protein molecules/AU | 2 protein molecules/AU |
| Solvent content | 52% | 48% |
|  |  |  |
| **Data Collection** |  |  |
| Wavelength | 0.9770 ^a^ | 0.9788 |
| Resolution (Å) | 2.24 (2.36-2.24) ^a^ | 2.84 (2.91-2.84) |
| Unique reflections | 53,198 ^a^ | 30,587 |
| *R*_merge_ (%) ^b^ | 6.9 (46) ^a^ | 7.1 (59.6) |
| *I*/σ ^c^ | 6.7 (1.5) ^a^ | 10.3.7 (1.8) |
| Completeness (%) | 99.0 (98.7) ^a^ | 99.4 (99.1) |
| Redundancy | 4.1 (3.9) ^a^ | 4.2 (3.9) |
|  |  |  |
| **Refinement** |  |  |
| Resolution (Å) | 32.80-2.24 | 46.01-2.84 |
| No. reflections | 61,706 | 30,587 |
| *R*_work_ / *R*_free_ (%) ^d^ | 18.4 / 21.2 | 22.7 / 26.8 |
| No. atoms |  |  |
| Protein | 8290 | 8257 |
| Ligand/ion | 90 | 0 |
| Water | 578 | 240 |
| *B*-factors (Å^2^) Overall / Wilson | 55.0 / 42.7 | 100.0 / 70.1 |
| R.m.s. deviations |  |  |
| Bond lengths (Å) | 0.010 | 0.010 |
| Bond angles (°) | 1.04 | 1.20 |

AU, asymmetric unit; r.m.s., root-mean-square deviation. Values in parentheses indicate the corresponding statistics in the highest-resolution shell.

^a^ from ([1](#_ENREF_1))

^b^ *R*_merge_ = Σ_hkl_ Σ_i_ | I_i_(hkl) − 〈I(hkl)〉|/ Σ_hkl_ Σ_i_I_i_(hkl), where I_i_(hkl) is the ith observation of reflection hkl and 〈I(hkl)〉 is the weighted average intensity for all observations i of reflection hkl.

^c^ *I*/σ is the mean signal-to-noise ratio, where I is the integrated intensity of a measured reflection and *I* is the estimated error in the measurement.

^d^ *R* = Σ_hkl_(||F_obs_|-|F_calc_||)/Σ_hkl_|F_obs_|, where |F_obs_| and |F_calc_| are the observed and calculated structure factor amplitudes, respectively.

**Supplementary Table 2. Root mean square deviations between the mimiviruses (Mg561 and R341) and the Vacccinia virus (VP55, PDB 2GA9) PAPs.**

|  |  | Megavirus Mg561 | | | | | | Mimivirus R341 | | | | | | Vaccinia virus VP55 | | |
| --- | --- | --- | --- | --- | --- | --- | --- | --- | --- | --- | --- | --- | --- | --- | --- | --- |
|  |  | Chain B | D1B | D2B | D3A | D3B | D4B | Chain A | Chain B | D1A | D2A | D3A | D4A | Full-length | D2 | D3 |
| Megavirus Mg561 | Chain A | 0.702 (487) |  |  |  |  |  | 1.257 (474) | 1.192 (479) |  |  |  |  | 2.59 (211) |  |  |
|  | D1A |  | 0.347 (36) |  |  |  |  |  |  | 0.772 (36) |  |  |  |  |  |  |
|  | D2A |  |  | 0.43 (162) | 2.029 (131) | 2.06 (132) |  |  |  |  | 0.928 (158) | 2.237 (129) |  |  | 2.094 (120) | 5.238 (66) |
|  | D3A |  |  | 2.237 (132) |  | 0.365 (209) |  |  |  |  | 2.161 (137) | 0.785 (172) |  |  | 2.293 (132) | 2.482 (91) |
|  | D4A |  |  |  |  |  | 0.976 (78) |  |  |  |  |  | 1.777 (101) |  |  |  |
| Mimivirus R341 | Chain A | 1.303 (472) |  |  |  |  |  |  | 0.692 (492) |  |  |  |  |  |  |  |
|  | Chain B | 1.237 (479) |  |  |  |  |  | 0.692 (492) |  |  |  |  |  |  |  |  |
|  | D2A |  |  |  |  |  |  |  |  |  |  | 2.874 (150) |  |  | 2.079 (119) | 4.266 (100) |
|  | D3A |  |  |  |  |  |  |  |  |  | 2.874 (150) |  |  |  | 3.426 (151) | 3.932 (113) |
| Vaccinia Virus VP55 | D2 |  |  |  |  |  |  |  |  |  |  |  |  |  |  | 2.551 (91) |

RMSD values (Å) were measured after superimposition of the whole proteins or domains with the UCSF Chimera software. The number of superimposed Cα is given in parenthesis.

**
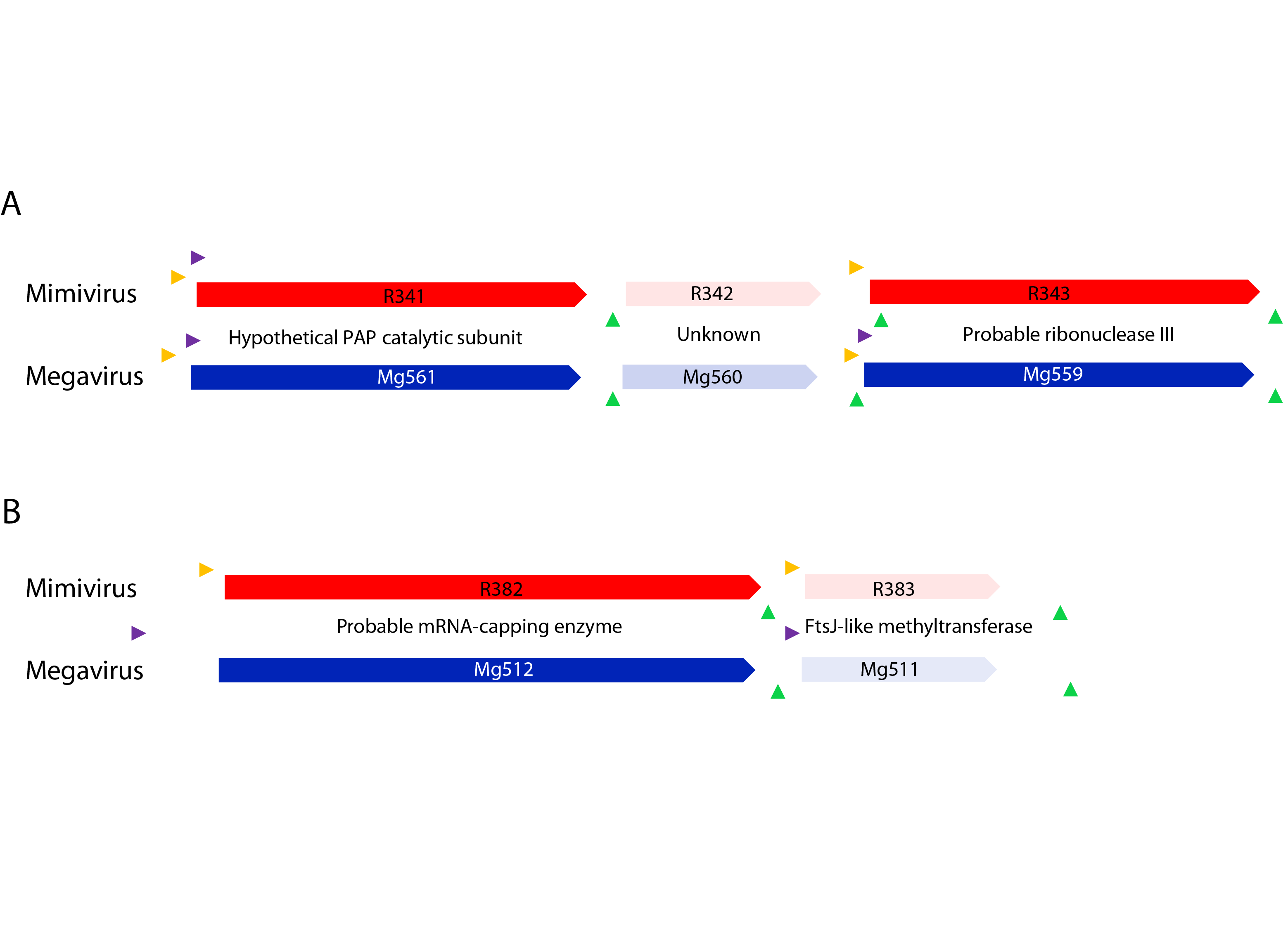
**

**
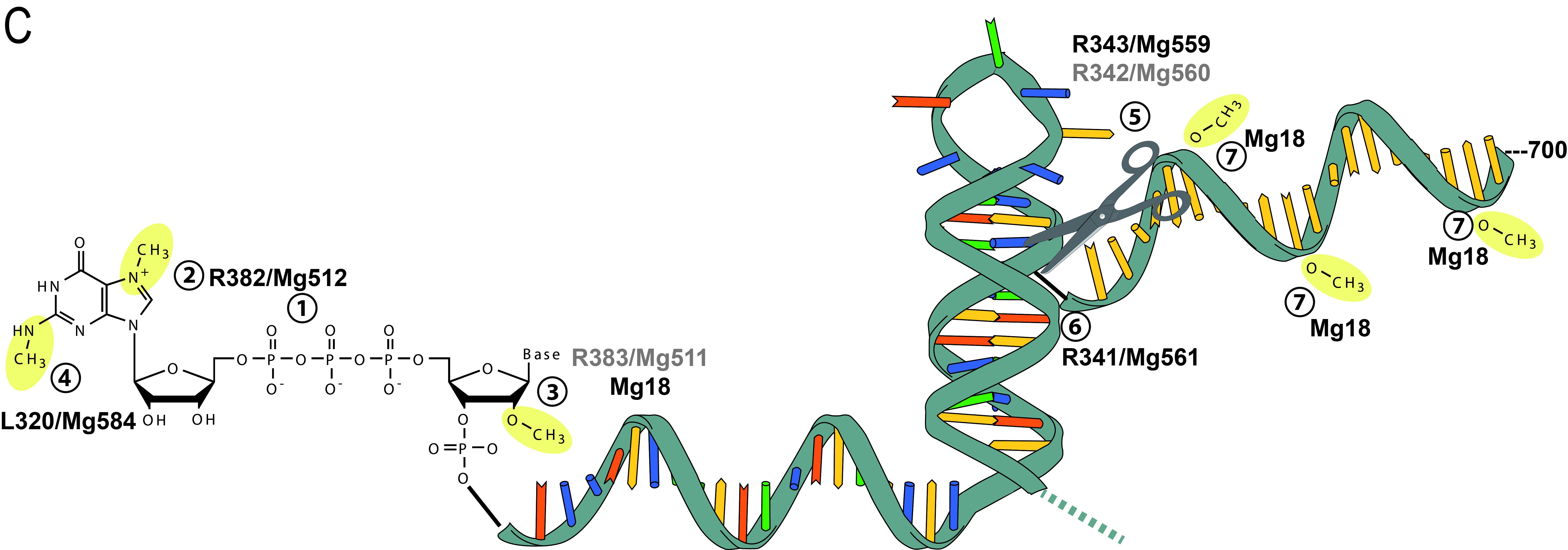
Supplementary Figure S1. Organisation of the conserved gene clusters**

Enzymes responsible for the viral mRNA 3’-end maturation (A), and for the 5’-end maturation (B). Genes corresponding to uncharacterized enzymes are in transparent. The hairpins (green triangles) and promoters (early, in orange and late in purple) are marked on the figure. (C) Schematic representation of the 7 steps and the viral enzymes involved in the mRNA transcript maturation. Step 1 corresponds to the capping, step 2 to the N7 methylation, step 3 to the cap 2’O methylation, step 4 to the cap N2 methylation, step 5 the hairpin recognition and cleavage, step 6 to the polyadenylation and step 7 to the internal 2’O methylation.

**Supplementary Figure S2. Multiple sequence alignment of viral PAPs sequences.**

Structural alignment of available viral PAPs sequences using the 3D-coffee server ([2](#_ENREF_2)) and manual editing. Helices are shown as coils and beta strands are shown as arrows on top of the multiple alignment. The red stars highlights the conserved NT signature G[G/S]x_n_Dx[D/E], and the orange stars the corresponding residues in the NT-like domain. The figure was prepared using ESPript server (<http://espript.ibcp.fr>) ([3](#_ENREF_3)). Sequences accession numbers: PAP_MEGA YP_004894612 , PAP_MIMI YP_003986843, PAP_MOUMOU AEX62700, PAP_CROV YP_003969918, PAP_OLPV1 ADX05881, PAP_OLPV2 ADX06298, PAP_PGV YP_008052392, PAP_ASFAR NP_042761, PAP_Vaccinia YP_232939. The secondary structure elements of each domain are colour coded as in Figure 1.

**Supplementary Figure S3. Kinetic analysis of binding of Mg18 to Mg561 and R341 by BioLayer Interferometry (BLI).**

Mg18 was immobilized on NiNTA BLI biosensors. (A) Increasing concentration ranging from 0.67 to 13.48 μM of Mg561 were tested for K_D_ determination. (B) The binding properties of Mg18 were assessed using Mg561 and R341 at 6µM.

**
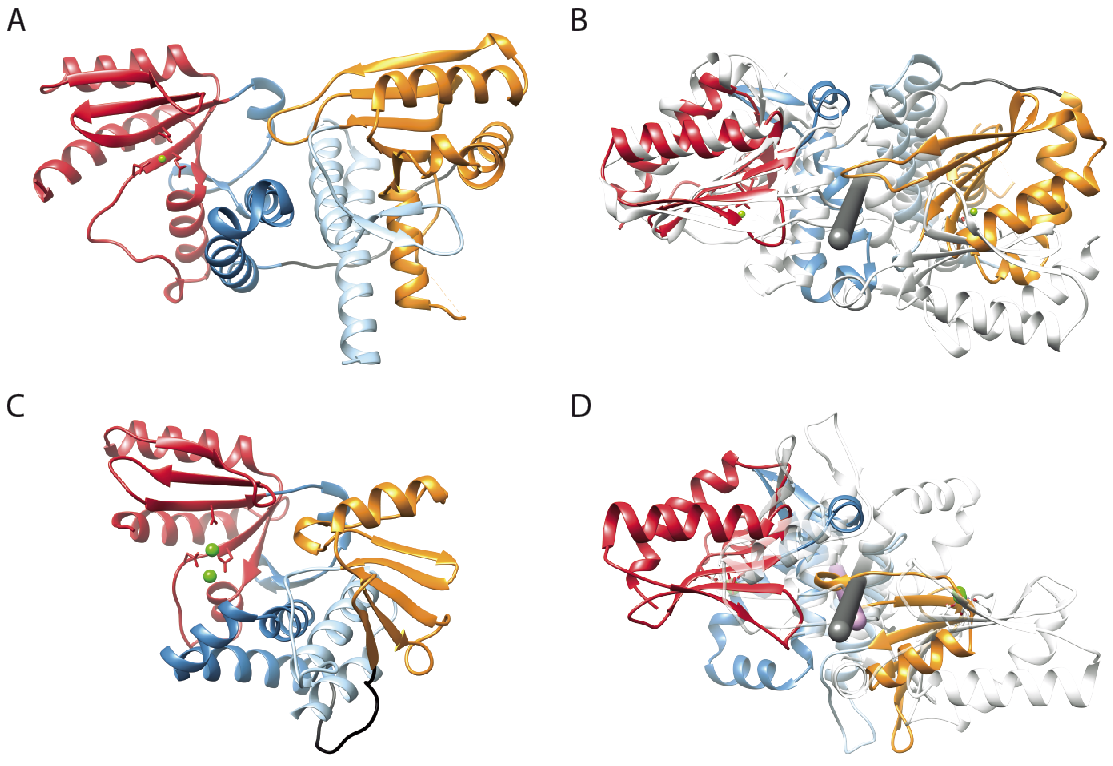
**

**Supplementary Figure S4. Internal symmetry of the viral PAPs.**

(A) Ribbon representation of the D2 and the D3 domain of Mg561. The water molecule at the cation location is marked as a green ball (B) Superimposition of D3 domain of monomer B (grey) on the D2 domain of A (red). The grey pole represents the 162° rotation and 2.4Å translation applied for superimposition. (C) Ribbon representation of the D2 and the D3 domains of Vaccinia virus VP55 in the same orientation than in (A). (D) Superimposition of the D3 domain on the D2 domain of VP55, the grey pole representing a 179° rotation and 5Å translation. Ca^2+^ ions are presented as green balls.


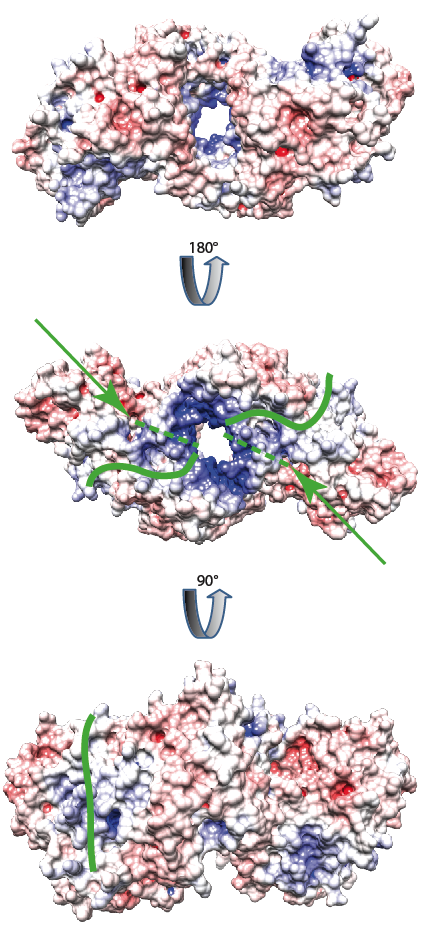


**Supplementary Figure S5. Electrostatic surface representation of the R341 protein.**

The R341 dimer was represented in the same orientation than in Figure 4. Contour levels: red, V <-10 kcal/mol; white, -10kcal/mol <V<+10kcal/mol; blue, V>+10 kcal/mol.


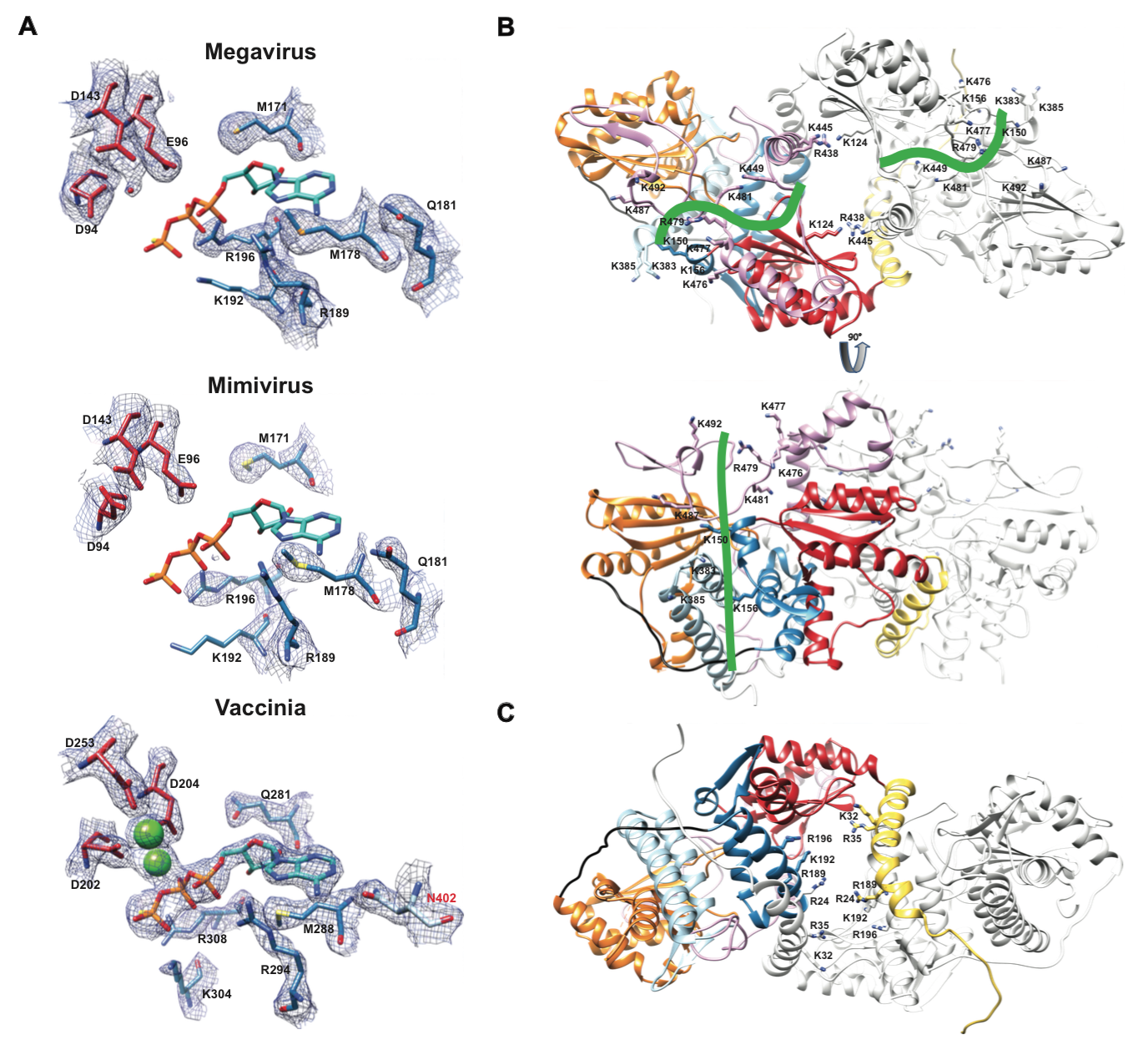


**Supplementary Figure S6. Mimiviruses PAPs active site compared to VP55 and proposed RNA binding and PPi efflux paths.**

(A) Close-up view of the catalytic sites with the structurally equivalent residues in Mg561 (upper), R341 (middle) and VP55 (bottom) in the same orientations. The figure was produced using the UCSF chimera software ([4](#_ENREF_4)). The VP55 residues involved in phosphates interaction (K_304_ and R_308_) and adenine selection (M_288_ and R_294_) ([5](#_ENREF_5)) are conserved in Mimiviruses PAPs (K_192_, R_196_ and M_178_, R_189_, respectively). The main chain carbonyl oxygen of Q_281_ of VP55, previously shown to make hydrogen bonds with the ribose 2’OH of ATP is replaced by M_171_ in the Mg561 active site. All ATP-interacting residues belong to the D2 subdomain except for N_402_, highlighted in red. The 2Fo-Fc electronic density maps are contoured at 1 σ. The 2GA9 calcium atoms are marked as green balls, the water molecule at the cation location in Mg561 structure is marked as a red ball (B). Proposed mimiviruses PAP RNA binding mode. The green lines correspond to the possible RNA binding sites in the positively charged grooves shown in Figure 5A. Arginine and Lysine residues in the positively charged grooves are shown. (C). Proposed mimiviruses PAP PPi outlet mode. Arginine and Lysine residues located at the central tunnel exit are shown.


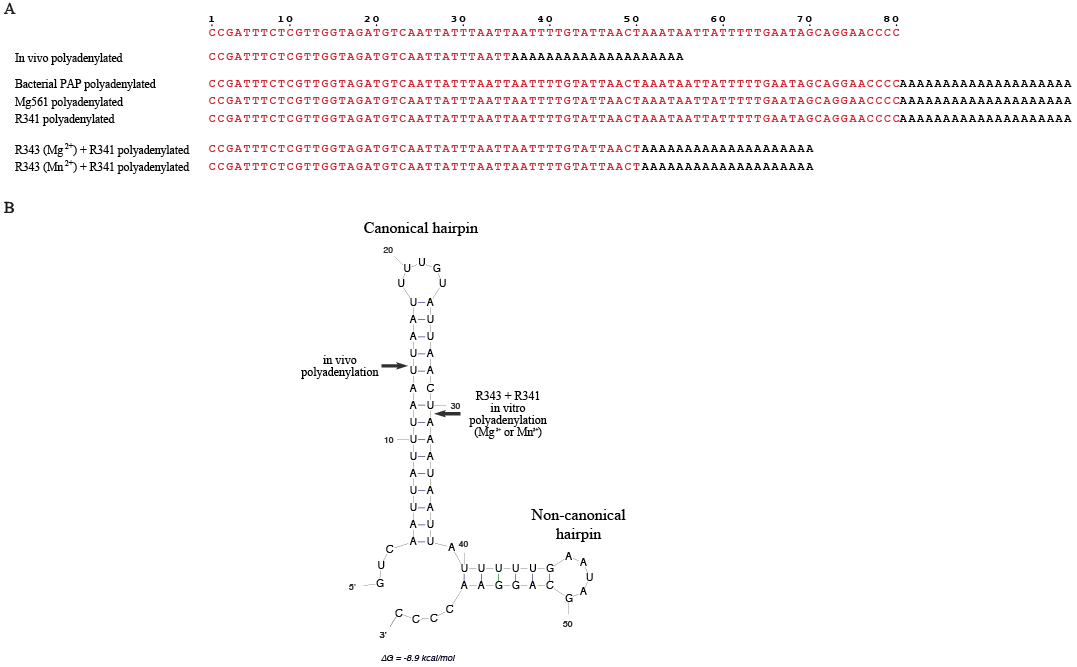


**Supplementary Figure S7. *In vivo* and *in vitro* Polyadenylation sites in a natural hairpin.**

(A) The M. chilensis transcripts of the *Mg592* gene with its 3’UTR hairpin was used in an *in vitro* polyadenylation assay with the Mimiviruses PAPs (Mg561 and R341). Where indicated the RNA were preincubated with the RNase III-like R343 in the presence of either Mg^2+^ or Mn^2+^. The bacterial PAP was used as a control. Polyadenylated products were purified and sequenced after reverse transcription. The *Mg592* RNA transcripts produced in *A. castellanii* 4 hours post-infection by *M. chilensis* were also sequenced. (B) Secondary structure prediction (Mfold server ([6](#_ENREF_6)) of the 3’ hairpin of the RNA used in (A). Cleavage sites are marked by arrows.

**Supplementary Figure S8. AdoMet-dependent MTase activity on short capped RNA.**

AdoMet-dependent MTase assays were performed on short capped RNA substrates GpppAN_13_, N7-methylated capped RNA substrates ^7Me^GpppAN_13_ and 2’O-methylated capped RNA substrates GpppA_2’OMe_N_13_. The methyl transfer to the capped RNA substrates was measured in the presence of [^3^H]-AdoMet for all viral PAPs, as well as for Mg18 incubated with Mimiviruses PAPs.

**References**

1. Lartigue, A., Jeudy, S., Bertaux, L. and Abergel, C. (2013) Preliminary crystallographic analysis of a polyadenylate synthase from Megavirus. *Acta crystallographica. Section F, Structural biology and crystallization communications*, 69, 53-56.

2. Poirot, O., Suhre, K., Abergel, C., O'Toole, E. and Notredame, C. (2004) 3DCoffee@igs: a web server for combining sequences and structures into a multiple sequence alignment. *Nucleic acids research*, 32, W37-40.

3. Robert, X. and Gouet, P. (2014) Deciphering key features in protein structures with the new ENDscript server. *Nucleic acids research*, 42, W320-324.

4. Pettersen, E.F., Goddard, T.D., Huang, C.C., Couch, G.S., Greenblatt, D.M., Meng, E.C. and Ferrin, T.E. (2004) UCSF Chimera--a visualization system for exploratory research and analysis. *Journal of computational chemistry*, 25, 1605-1612.

5. Moure, C.M., Bowman, B.R., Gershon, P.D. and Quiocho, F.A. (2006) Crystal structures of the vaccinia virus polyadenylate polymerase heterodimer: insights into ATP selectivity and processivity. *Molecular cell*, 22, 339-349.

6. Zuker, M. (2003) Mfold web server for nucleic acid folding and hybridization prediction. *Nucleic acids research*, 31, 3406-3415.
